# Reference Gene Selection for Normalizing Gene Expression in *Ips Sexdentatus* (Coleoptera: Curculionidae: Scolytinae) Under Different Experimental Conditions

**DOI:** 10.3389/fphys.2021.752768

**Published:** 2021-10-27

**Authors:** Gothandapani Sellamuthu, Shan Amin, Jan Bílý, Jirí Synek, Roman Modlinger, Madhab Kumar Sen, Amrita Chakraborty, Amit Roy

**Affiliations:** ^1^Excellent Team for Mitigation (ETM), Faculty of Forestry and Wood Sciences, Czech University of Life Sciences Prague, Prague, Czechia; ^2^Department of Biology, Lund University, Lund, Sweden; ^3^Department of Agroecology and Crop Production, Faculty of Agrobiology, Food and Natural Resources, Czech University of Life Sciences Prague, Prague, Czechia; ^4^EVA 4.0 Unit, Faculty of Forestry and Wood Sciences, Czech University of Life Sciences Prague, Prague, Czechia

**Keywords:** *Ips sexdentatus*, reference gene, RT-qPCR, differential gene expression, housekeeping genes, bark beetles, Scolytinae

## Abstract

*Ips sexdentatus* (Coleoptera: Curculionidae: Scolytinae) is one of the most destructive and economically important forest pests. A better understanding of molecular mechanisms underlying its adaptation to toxic host compounds may unleash the potential for future management of this pest. Gene expression studies could be considered as one of the key experimental approaches for such purposes. A suitable reference gene selection is fundamental for quantitative gene expression analysis and functional genomics studies in *I. sexdentatus*. Twelve commonly used reference genes in Coleopterans were screened under different experimental conditions to obtain accurate and reliable normalization of gene expression data. The majority of the 12 reference genes showed a relatively stable expression pattern among developmental stages, tissue-specific, and sex-specific stages; however, some variabilities were observed during varied temperature incubation. Under developmental conditions, the *Tubulin beta-1 chain* (β*-Tubulin*) was the most stable reference gene, followed by *translation elongation factor* (*eEF2*) and *ribosomal protein S3* (*RPS3*). In sex-specific conditions, *RPS3*, β*-Tubulin*, and *eEF2* were the most stable reference genes. In contrast, different sets of genes were shown higher stability in terms of expression under tissue-specific conditions, i.e., *RPS3* and *eEF2* in head tissue, *V-ATPase-A* and *eEF2* in the fat body, *V-ATPase-A* and *eEF2* in the gut. Under varied temperatures, β*-Tubulin* and *V-ATPase-A* were most stable, whereas *ubiquitin (UbiQ)* and *V-ATPase-A* displayed the highest expression stability after Juvenile Hormone III treatment. The findings were validated further using real-time quantitative reverse transcription PCR (RT-qPCR)-based target gene expression analysis. Nevertheless, the present study delivers a catalog of reference genes under varied experimental conditions for the coleopteran forest pest *I. sexdentatus* and paves the way for future gene expression and functional genomic studies on this species.

## Introduction

Differential gene expression (DGE) studies are fundamental to evaluate the effects of physiological responses on biological variation in insect populations at the molecular level. Real-time quantitative reverse transcription PCR (RT-qPCR) is a reliable technique and is widely used to analyze the expression of target genes due to its high sensitivity, accuracy, specificity, reproducibility, and speed (Heid et al., 1996; Bustin et al., 2005; VanGuilder et al., 2008; Wang et al., 2020). In addition, RT-qPCR allows simultaneous measurement of gene expression in many different samples for a limited number of target genes and is particularly suitable when only a limited number of samples are available (Higuchi et al., 1993; Vandesompele et al., 2002). Nevertheless, expression results vary due to initial sample size, RNA integrity (quantity and quality), reverse transcription, messenger RNA (mRNA) recovery, PCR efficiency, and primer design (Gao et al., 2020). Therefore, internal control genes (alternatively called reference genes) are commonly used to normalize mRNA levels for more accurate gene expression quantification as their expression should not vary under different biological or experimental conditions (Nicot et al., 2005; Lu et al., 2018). Furthermore, it is strongly recommended to use multiple reference genes for target gene expression normalization for more authenticity (García-Reina et al., 2018; Shakeel et al., 2018; Wang et al., 2020). There are several studies on gene function analysis in insects using reference genes such as β-actin (*Actin*), glyceraldehyde-3-phosphate dehydrogenase (*GAPDH*), elongation factor 1α (*EF1A*), β-Tubulin (β*-Tubulin*), ribosomal proteins (*RPs*), ubiquitin (*UbiQ*), superoxide dismutase (*SOD*), heat shock protein 90 (*HSP90*), and vacuolar-type H+-ATPase subunit B (*V-ATPase-B*) for gene expression normalization (Lu et al., 2018; Qu et al., 2018; Gao et al., 2020; Wang et al., 2020). This suggests that reference genes can be differentially expressed in insects under varied experimental conditions. Alternatively, no universal reference gene is available, which is stably expressed under different experimental conditions (Lu et al., 2018). Therefore, identification and validation of reference genes under different experimental conditions, life stages, and tissue-specific stages are essential on a case-by-case basis for accurate quantification of gene expression in any insects, including *I. sexdentatus* (Pfaffl et al., 2004; Rodrigues et al., 2014; Qu et al., 2018; Basu et al., 2019; Gao et al., 2020; Gurusamy et al., 2021; Xie et al., 2021).

The wood-boring insect *I. sexdentatus* (Coleoptera: Curculionidae: Scolytinae; hereby referred to as ISx), also known as the six-toothed bark beetle, is one of the most destructive and economically important insects, causing severe damage to coniferous species throughout Europe and Asia (Etxebeste and Pajares, 2011; Seidl et al., 2017; Douglas et al., 2019). ISx is an opportunistically aggressive species of bark beetles (Wermelinger et al., 2008; Chakraborty et al., 2020a). It usually attacks old scots pine trees and can occupy young trees up to a diameter of 10 cm in dbh (diameter at breast height). It was primarily a sparse specimen (Pfeffer, 1955; Postner, 1974) in the past, but now ISx is considered a significant pest in some European countries (Gregoire and Evans, 2004). Several drought periods during the last decade highly favored its current upliftment on the pest status, and we are registering higher population densities of ISx in most of the Czech forests. At least from 2018, the south Moravian forests suffered from the severe outbreak of this species. The volume of the harvested pine due to bark beetles in the most affected region was 12 times higher than in the previous years (Lubojacký and Knížek, 2020).

Bark beetles preferentially colonize weakened, wilted, or recently dead trees. However, favorable conditions such as drought can lead to an outbreak (epidemic phase). Beetles start attacking the healthy standing trees due to weakened defenses caused by warmer temperatures (Bouhot et al., 1988; Marini et al., 2017; Pettit et al., 2020) and larger windthrows (Kausrud et al., 2012; Biedermann et al., 2019; Sommerfeld et al., 2020). Furthermore, conifers have self-defense mechanisms to ward off bark beetle infestations with secondary metabolites such as terpenes (Ferrenberg et al., 2014; Denham et al., 2019). These defense mechanisms can also be overcome during mass insect attacks by detoxifying plant-derived secondary metabolites with the help of symbiotic microbes (Chakraborty et al., 2020b; Huang et al., 2020). To elucidate molecular underpinnings shaping environment-conifer-bark beetle (i.e., *I. sexdentatus*) interactions, gene expression studies need to be conducted in the future. Such studies advocate the requirements for stable reference genes under different experimental conditions.

Hence, we extensively evaluated 12 commonly known reference genes in Colepterans and other insects (Supplementary Figure 1 and Supplementary Table 1) for the expression stability in ISx under varied experimental conditions in the present study. Using available in-house transcriptome data of ISx, we have obtained gene sequences of β*-actin* (*Actin*), *translation EF 2* (*eEF2*), *Tubulin beta-1 chain* (β*-Tubulin*), *myosin regulatory light chain 2* (*Myosin L*), *V-type proton ATPase catalytic subunit A* (*V-ATPase-A*), *NADH dehydrogenase subunit 1* (*NADH*), *ubiquitin C variant* (UbiQ), *glyceraldehyde-3-phosphate dehydrogenase* (*GAPDH*), *arginine kinase isoform X1*(*ArgK*), *RPS3, RPL17*, and *HSP83* for expression stability evaluation. The candidate reference genes were screened using different parameters, such as developmental stages (i.e., larvae, pupae, and adult stages), tissue- and sex-specific [head, gut, fat body, and whole body (WB) except the head, gut, fat body], and different treatments [such as temperature; Juvenile hormone III (JHIII); wild-collected vs. long-term laboratory-reared; and different host feeding] to obtain accurate reference genes for future genomic and functional studies. Our study delivers a catalog of genes that should be used for DGE studies on ISx under varied experimental conditions and commences the possibility for aggressive management of bark beetles using molecular approaches such as RNA interference (RNAi) (Joga et al., 2021).

## Materials and Methods

### Bark Beetles

*Ips sexdentatus* (ISx)-infested *Pinus sylvestris* were collected from Kostelec nad Cernými lesy (50°00'07.2”N 14°50'56.3”E, under School Forest Enterprise) maintained insect rearing chambers at Faculty of Forestry and Wood Sciences, Czech University of Life Sciences, Prague. Beetles were maintained with fresh pine logs at 27 ± 1°C under 70 ± 5% humidity and a 16:8-h light/dark (L:D) photoperiod. ISx is the largest beetle of the genus *Ips*, at 6-8 mm in length. The life stages include three larval stages, pupae, callow (just emerged), and fed adult stage (flying for new host colonization). Both sexes have six spines at each side of the elytral declivity. The fourth spine is the largest and is capitate. The wild population was supplied with fresh pine logs for the next generation and maintained for 40–45 days for one life cycle. Similarly, the wild population was continuously maintained for three generations to study the difference between wild and lab-reared beetle conditions.

### Sample Preparation

#### Development Stages and Different Tissue Types

Samples were collected from different life stages of ISx: three larval stages (L1, L2, and L3), pupae, (P), callow (newly emerged) male (CM) and female (CF), and fed adult (mature adults move toward trunks of pines to lay eggs for next-generation) male (AMF) and female (AFF). For sufficient nucleic acid extraction for downstream processing, each biological replicate was prepared using pooled individuals from various developmental stages, such as five first instar larvae (L1)/replicate, three second and third instar larvae (L2 and L3)/ replicate, three pupae/ replicate, and two adults/replicate. Four biological replicates were used in all experiments.

Tissues such as head, fat body, gut, and WB minus head, fat body, and gut (hereby referred to as WB) were dissected from the callow and fed adult stage of both male and female ISx producing experimental samples, such as CMH, callow male beetle head; CMFB, callow male beetle fat body; CMMG, callow male beetle midgut; CMWB, callow male beetle WB; CFH, callow female beetle head; CFFB, callow female beetle fat body; CFMG, callow female beetle midgut; CFWB, callow female beetle WB; and AMFH, fed adult male head; AMFFB, fed adult male fat body; AMFMG, fed adult male midgut; AMFWB, fed adult male WB; AFFH, fed adult female head; AFFFB, fed adult female fat body; AFFMG, fed adult female midgut; and AFFWB, fed adult female WB. Four independent biological replicates were collected and stored at −80°C. Each replicate was derived by pooling tissues from 10 individual beetles.

#### Temperature and JHIII Treatment

For the temperature treatment, the freshly emerged adults were placed in small glass tubes and exposed to a range of temperatures (4, 27, and 37°C) for 72 h in a temperature-controlled chamber. After exposure, survivors were frozen in liquid nitrogen and stored at −80°C. Adults maintained at 27°C were used as control. At least four beetles were randomly collected per replication, and four independent biological replications were used from each temperature treatment.

For JHIII treatment, emerged beetles were sorted based on gender and kept at 4°C on moist paper towels. Beetles were treated topically on the ventral surface of the abdomen with 10 μg JHIII (dissolved in acetone) (Sigma-Aldrich, St. Louis, MO, USA) and only acetone as control (Aw et al., 2010; Sun et al., 2021). The beetles were incubated at 27 ± 1°C under 70 ± 5% humidity and a 16:8-h light/dark (L:D) photoperiod in groups of 20 in 60 ml plastic containers for 72 h. After incubation, beetles were immediately shock frozen in liquid nitrogen and stored at −80°C for downstream processing. Four biological replicates were used for JHIII treatment and control.

#### Different Host Feeding

Under different host feeding treatments, ISx was placed into the phloem tissue of freshly cut lodgepole pine and spruce. Beetles were placed under the bark in randomly chosen male-female pairs and maintained under standard rearing conditions. ISx was allowed to feed under the bark for one generation (40–45 days). We removed ISx adults from galleries showing the excavation of frass (an indication of feeding). The beetles were collected and immersed in insect ringer solution; the gut tissues were excised. Gut tissues were gently purged of their contents (i.e., malpighian tubules, fat body) and then frozen in liquid nitrogen and stored at −80°C.

#### Total RNA Extraction and Complementary DNA Synthesis

Total RNA from bark beetle tissue samples was extracted using TRIzol® (Invitrogen, Carlsbad, CA) following the protocol of the manufacturer. Isolated RNA was further treated with DNases using a TURBO DNAase Kit (Ambion, USA) to remove any DNA contamination. RNA quantity and quality were evaluated using 1.5% agarose gel and quantified by NanoDrop 2000/2000c from Thermo Fisher Scientific® (Waltham, MA, USA) and stored at −80°C. Complementary DNA (cDNA) (first-strand) was synthesized from 1 μg of total RNA in triplicate using the high-capacity cDNA reverse transcription kits (Applied Biosystems-Life Technologies) following the recommendations of the manufacturer and stored at −20°C.

### Selection of Candidate Reference Genes for Evaluation

This study selected 12 potential reference genes (Supplementary Table 2) that function as reference genes in other Coleoptera species (Supplementary Figure 1 and Supplementary Table 1). These genes were obtained from our functionally annotated in-house transcriptome of ISx (manuscript in preparation). The primers for those selected genes were designed *via* Integrated DNA Technologies, Inc. (IDT) (Table 1).

**Table 1:** Details of primer sequence, amplicon length, and RT-qPCR analysis of candidate reference genes and target genes.

**Gene symbol**	**Gene name**	**Primer sequences (5^**′**^-3^**′**^)**	**Amplicon length (bp)**	**PCR efficiency %**	**Regression coefficient**
*Actin*	β-actin	**FP**: GCATACGGTCAGCAATACC	118	101.14	0.988
		**RP**: CACGAAACCGTCTACAACTC			
*eEF2*	Translation elongation factor	**FP**: CGATGGCCTCAACGTAAC	142	101.30	0.981
		**RP**: CGTGTGTTCTCCGGTAAAG			
*β-Tubulin*	Tubulin beta-1 chain	**FP**: CATCTCGTCCATACCCTCT	126	105.85	0.987
		**RP**: GCCACCTTCATCGGTAAC			
*Myosin L*	Myosin regulatory light chain 2	**FP**: GACCCAACAACTGGGTAAG	140	98.69	0.990
		**RP**: CCACGACAAAGACGGTATC			
*V-ATPase-A*	V-type proton ATPase catalytic subunit A	**FP**: CGTTTGGCCTCCTTCTAC	125	110.71	0.9999
		**RP**: GTGACAGGATCGGAGAAATC			
*NADH*	NADH dehydrogenase subunit 1	**FP**: GTGTGAGTGCAGACCAAC	148	108.07	0.9941
		**RP**: GAACGAGAGCCGAAGAAAC			
*UbiQ*	Ubiquitin C variant	**FP**: TGAGGCTAAGAGGAGGAATG	120	98.17	0.989
		**RP**: CGTCCTTGTCCTGGATCT			
*GAPDH*	Glyceraldehyde-3-phosphate dehydrogenase	**FP**: CACCCAGAAGACTGTTGAC	129	107.06	0.977
		**RP**: CCGTTCAGGGAAGGAATAAC			
*ArgK*	Arginine kinase isoform X1	**FP**: CCGTCTTCTCTGACTTGTTC	144	106.49	0.9802
		**RP**: GGGTGGACACAACGTATTC			
*RPS3*	Ribosomal protein S3	**FP**: GAACGGACCGTGTTCTTG	128	98.32	0.973
		**RP**: CAGACCTCTGTTGACTACCT			
*RPL17*	60S ribosomal protein L17	**FP**: ACGTGCACTTCACACTTC	102	106.54	0.9895
		**RP**: TCCTCACCTCTCACCTAAAG			
*HSP83*	Heat shock protein 83	**FP**: GCTTTCTGGCGTAGGTTT	138	110.00	0.974
		**RP**: CGGATGGACTGCCAATATG			
**Target gene**					
*Kr-h1*	Kruppel homolog 1	**FP:** GCTGTTCTCAATTCTCCATGC	168	113.9	0.833
		**RP**: ACGGTGTAAGGCATCAGAATG			
*Hsp70*	Heat shock protein 70	**FP:** GACTATGGGTATTGAGACTGTG	140	104.9	0.961
		**RP**: CACCTTCGTACACCTGAATAG			

### Quantitative RT-qPCR Analysis

cDNA samples were diluted 1:20 before being used in RT-qPCR. Four independent biological replicates from each treatment, developmental stage, and tissue-type were included in each RT-qPCR run. RT-qPCR run was performed using the Applied Biosystems™ StepOne™ Real-Time PCR System (Applied Biosystems) with a reaction mix containing 5.0 μL of SYBR® Green PCR Master Mix (Applied Biosystems), 1.0 μL of cDNA, 1.0 μL optimized concentrations of primers (Table 1), and RNase-free water (Invitrogen) to a total volume of 10.0 μL. Amplification conditions were as follows: initial denaturation at 95°C for 10 min, followed by 40 cycles of 95°C for 15 s, and 60°C for 1 min. In order to confirm the primer specificity, we performed melt curve analysis using default parameters by a steady increase in temperature from 60 to 95°C. All RT-qPCR assays were carried out in four biological replicates, including two or three technical replicates.

### Data Analyses

The raw cycle threshold (Ct) values were obtained with 7500 software v2.0.5 (Applied Biosystems®). Gene expression and stability were analyzed using four different commonly used tools and algorithms such as geNorm (Vandesompele et al., 2002), BestKeeper (Pfaffl et al., 2004), NormFinder (Andersen et al., 2004), and ΔCt method (Silver et al., 2006).

Precisely, geNorm assesses the expression stability value (M) as the average pairwise variation of one of the genes against all control genes present in the experiment. The program estimates the mean pairwise variation between genes across all samples, and the gene with the lowest M value is considered most stable (Vandesompele et al., 2002). NormFinder calculates the SD for each target gene and juxtaposes it with other gene expressions. The gene displaying the lowest variation between intra- and inter-group comparisons is considered most stable (Andersen et al., 2004). On the contrary, BestKeeper is a data processing method based on crossing points that compares all genes across all samples and provides a stability index for each reference gene (Pfaffl et al., 2004). The comparative delta-Ct method juxtaposes Ct values and the relative expression of “gene pairs” within each sample (Silver et al., 2006). The sample-specific mean Ct values of each reference gene from each experiment are given as input data and subsequently processed using the web-based tool RefFinder (https://www.heartcure.com.au/reffinder/).

Furthermore, pairwise variation (V), estimated by geNorm, was used to decide the optimal number of reference genes for accurate RT-qPCR normalization. The Vn/Vn+1 value reflected the pairwise variation between two sequential normalization factors. A cutoff threshold, i.e., Vn/Vn+1 = 0.15, was set for a valid normalization (Vandesompele et al., 2002).

### Validation of Selected Reference Genes

We analyzed the relative expression levels of *Kr-h1* and *Hsp70* genes after JHIII treatment and different temperature exposure to evaluate the selected reference genes. To compare the impact of different normalization strategies, the expression of target genes was normalized using both selected reference genes individually and in combinations using the 2^−ΔΔCt^ method (Livak and Schmittgen, 2001). Target genes expression were analyzed using one-way ANOVA using GraphPad Prism software. *P*-value < 0.05 was considered to identify significant differences between samples.

## Results

### Primer Specificity and PCR Efficiency

**Figure 1:**
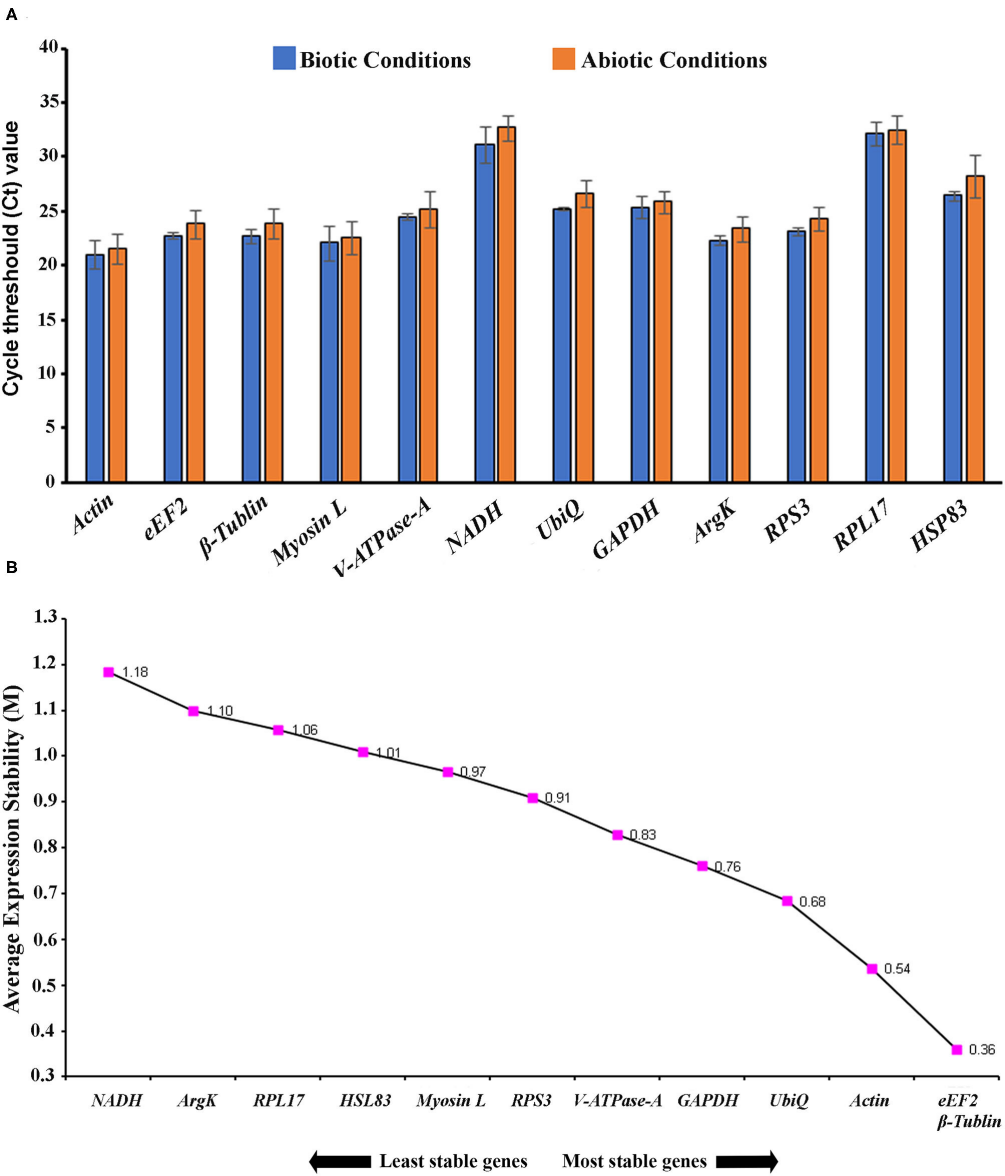
**(A)** Expression range of cycle threshold (Ct) values of candidate reference genes under different experimental conditions in *I. sexdentatus* (ISx). The different conditions included in biotic conditions: developmental stages (i.e., larvae, pupae, and adult stages), sex-specific (male and female), tissue types [head, midgut, fat body, and whole body (WB) except head, gut, fat body (Abdomen)] and abiotic conditions: different treatments (such as temperature; Juvenile hormone III; wild-collected vs. long-term laboratory-reared; and different host feeding). **(B)** The average expression stability values (M) of 12 reference genes in different developmental stages (larvae, pupae, and adult stages) were plotted from the least stable (left) to the most stable (right).

In the current study, 12 genes, namely *Actin, eEF2*, β*-Tubulin, Myosin L, V-ATPase-A, NADH, UbiQ, GAPDH, ArgK, RPS3, RPL17*, and *HSP83*, were screened for identifying suitable reference gene or gene combination from ISx. RT-qPCR products generated with each primer set (forward+ reverse) against target genes were evaluated by the occurrence of a single peak in melting curve analyses (Supplementary Figure 2) and specific bands of the expected size in agarose gel electrophoresis (Supplementary Figure 3). The amplification efficiency for each primer pair ranged from 98.17 to 107.06%, and the correlation coefficients (*R*^2^) were greater than 0.98 (Table 1). The Ct values of the 12 candidate reference genes ranged from 19.87 to 34.78 and covered all experimental conditions (Figure 1A). While most Ct values ranged from 19 to 27, *Actin, eEF2*, β*-Tubulin*, and *RPS3* were the most abundant transcripts under almost all experimental conditions. The least frequently expressed reference genes were *NADH, RPL17*, and *HSP83*. The five remaining reference genes were expressed at moderate levels.

### Expression Stability of Putative Reference Genes Under Biotic Conditions

For identifying stable reference genes, four different algorithms (geNorm, NormFinder, BestKeeper, and delta-Ct) were deployed to evaluate the stability of candidate reference genes under different experimental conditions [(i.e., developmental stages, tissue-specific, sex-specific) and different treatments (JHIII; wild-collected vs. long-term laboratory-reared; and different host feeding)] by using the RefFinder web-based tool that ranks reference genes.

#### Reference Gene for Developmental Stages

For the developmental stages, the order of stability of the first four most stable genes, namely β*-Tubulin, eEF2, RPS3*, and *GADPH*, identified by four programs, was inconsistent (Table 2). The least stable genes nominated by four programs were *Actin, RPL17*, and *HSP83*. The most stable genes were β*-Tubulin, eEF2*, and *RPS3* determined based on Normfnder rankings of 0.343, 0.347, and 0.379, respectively (Table 2). As per RefFinder, the stability ranking of the reference genes from most stable to least stable across different developmental stages were as follows: *eEF2*, β*-Tubulin, Actin, UbiQ, GAPDH, V-ATPase-A, RPS3, Myosin L, HSP83, RPL17, ArgK*, and *NADH* (Figure 1B). RefFinder identified the top three candidates, *eEF2*, β*-Tubulin*, and *Actin*, across developmental stages by integrating the results from all four programs.

**Table 2:** Ranking of the candidate reference genes based on stability values performed by Delta Ct, BestKeeper, RefFinder, and NormFinder, in different developmental stages [i.e., larval stages (L1, L2, and L3), pupae (P), callow (newly emerged) male (CM), and female (CF)] and fed adult (mature adults move toward trunks of pines to lay eggs for next-generation) male (AMF) and female (AFF).

**Genes**	**ΔCt method**		**Best keeper**		**RefFinder**		**NormFinder**		**Recommended genes**
	**Stability**	**Rank**	**Stability**	**Rank**	**Stability**	**Rank**	**Stability**	**Rank**	
*β-Tubulin*	3.1	1	1.79	4	1.86	1	0.343	1	*β-Tubulin*,
*GAPDH*	3.19	2	1.89	6	3.94	5	0.388	5	*eEF2*
*eEF2*	3.24	3	1.85	5	3.08	3	0.374	2	and *RPS3*
*RPS3*	3.26	4	1.6	3	2.63	2	0.379	3	
*Myosin-L*	3.35	5	1.58	2	3.66	4	0.420	9	
*V-ATPase-A*	3.37	6	2.18	7	4.92	7	0.380	4	
*ArgK*	3.54	7	1.43	1	3.96	6	0.429	10	
*UbiQ*	4.15	8	2.5	8	8.24	8	0.389	6	
*NADH*	4.21	9	4.11	10	9.21	9	0.639	12	
*HSP83*	4.53	10	3.08	9	9.72	10	0.405	7	
*RPL17*	4.98	11	4.94	11	10.74	11	0.523	11	
*Actin*	8.45	12	7.62	12	12	12	0.415	8	

#### Reference Gene for Tissue Stages

##### Target Gene Expression in Male and Female Tissues

**Figure 2:**
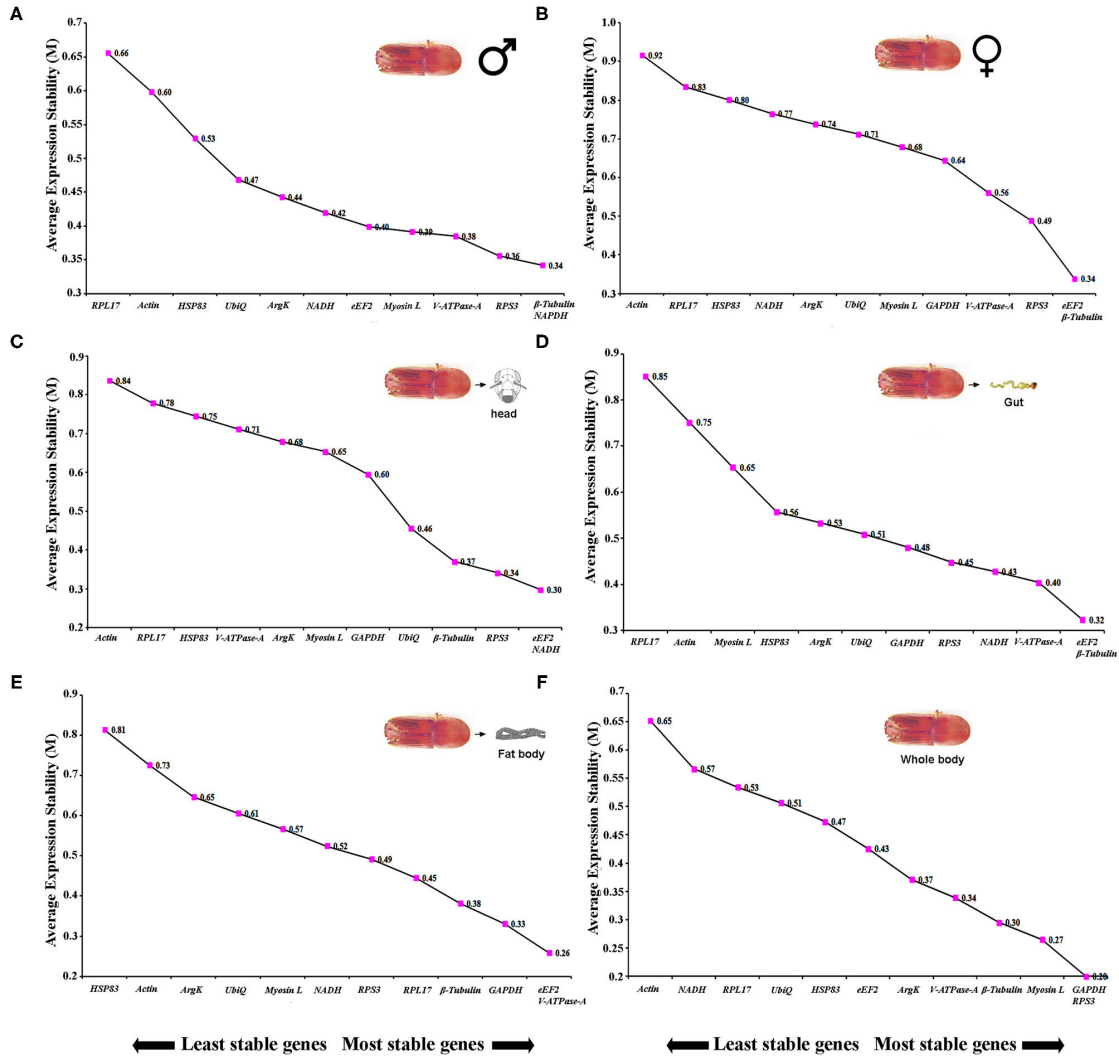
The average expression stability values (M) of 12 reference genes under different conditions (sex-specific and tissue types) were calculated by geNorm from the least stable (left) to the most stable (right). **(A)** male, **(B)** female, **(C)** head, **(D)** gut, **(E)** fat body, and **(F)** whole body (WB).

Sex-specific reference gene expressions were calculated separately for male (callow and fed adult) and female (callow and fed adult) insect tissues (i.e., head, fat body, gut, WB except for the head, fat body, and gut collected from male and female, or in short, abdomen). In male tissues, the order of stability of the first four most stable genes (*RPS3, GAPDH, eEF2*, and β*-Tubulin*) determined by four programs was inconsistent (Table 3). The least stable genes recommended by four programs were *Actin, RPL17*, and *Myosin L*. The stability ranking of the reference genes of the most stable and the top three candidates, β*-Tubulin, GAPDH*, and *RPS3*, was constant according to RefFinder (Figure 2A). In contrast, the female genes *eEF2, RPS3*, β*-Tubulin*, and *UbiQ* were the most stable genes (Table 3). The least stable gene was *ArgK*. The top three most stable reference genes, namely *eEF2*, β*-Tubulin*, and *RPS3*, were constant for females in the RefFinder analysis (Figure 2B).

**Table 3:** Ranking of the candidate reference genes based on stability values performed by Delta Ct, BestKeeper, RefFinder, and NormFinder, in sex-specific conditions (male and female).

**Conditions**	**Genes**	**ΔCt method**		**Best keeper**		**RefFinder**		**NormFinder**		**Recommended genes**
		**Stability**	**Rank**	**Stability**	**Rank**	**Stability**	**Rank**	**Stability**	**Rank**	
Male	*eEF2*	1.44	1	1.68	7	2.43	1	0.210	5	*RPS3, GAPDH* and *eEF2*
	*β-Tubulin*	1.6	2	1.95	8	4	5	0.145	2	
	*NADH*	1.63	3	1.34	5	3.41	4	0.274	9	
	*GAPDH*	1.63	4	2.15	10	6.16	7	0.145	1	
	*UbiQ*	1.66	5	1.12	3	4.16	6	0.210	6	
	*RPS3*	1.71	6	0.71	1	2.45	2	0.151	3	
	*ArgK*	1.79	7	0.79	2	3.15	3	0.234	8	
	*V-ATPase-A*	1.86	8	1.48	6	7.14	8	0.227	7	
	*HSP83*	1.89	9	2.08	9	8.97	10	0.294	10	
	*RPL17*	2.04	10	1.31	4	7.27	9	0.351	12	
	*Myosin L*	2.32	11	2.71	11	11	11	0.159	4	
	*Actin*	2.42	12	2.9	12	12	12	0.343	11	
Female	*eEF2*	1.31	1	1.5	4	1.41	1	0.255	1	*eEF2, RPS3* and *β-Tubulin*
	*RPS3*	1.44	2	1.17	2	1.86	2	0.266	3	
	*β-Tubulin*	1.51	3	1.48	3	4.05	4	0.274	6	
	*NADH*	1.63	4	1.63	8	5.26	6	0.373	4	
	*UbiQ*	1.64	5	1.09	1	2.94	3	0.350	10	
	*GAPDH*	1.67	6	2.08	10	7.33	8	0.273	5	
	*HSP83*	1.71	7	1.59	7	7	7	0.326	9	
	*RPL17*	1.78	8	1.17	2	4.76	5	0.402	11	
	*Myosin L*	1.91	9	2.48	12	9.93	10	0.265	2	
	*V-ATPase-A*	1.95	10	1.52	5	8.19	9	0.274	7	
	*Actin*	1.98	11	1.94	9	10.46	11	0.470	12	
	*ArgK*	2.02	12	2.17	11	11.74	12	0.325	8	

##### Target Gene Expression in Various Tissues

Tissue sections were obtained to evaluate the stability of candidate reference genes among different tissue (i.e., head, fat body, gut, and WB). In head tissues, the order of stability of the first four most stable genes (i.e., *RPS3, eEF2, ArgK*, and *V-ATPase-A*) recognized by four programs was inconsistent (Table 4). The least stable genes determined by four programs were *GAPDH, RPL17*, and *HSP83*. According to RefFinder, the stability ranking of reference genes from the most stable and the top three candidates were *eEF2, NADH*, and *RPS3* within head tissues (Figure 2C). Whereas among fat body tissues, *V-ATPase-A, eEF2, NADH*, and β*-Tubulin* genes were the most consistently expressed (Table 4). The least stable genes were *RPL17, Myosin L*, and *Actin*. The stability ranking (top three) of the reference genes was *eEF2*, β*-Tubulin*, and *V-ATPase-A* (Figure 2D) in the RefFinder analysis for the fat body. However, *V-ATPase-A, eEF2, RPL17*, and *NADH* were the most stable genes in the gut tissue (Table 4). The least stable genes in the gut were *HSP83, Myosin L*, and *Actin*. The final top three stable reference genes after RefFinder analysis were *eEF2, V-ATPase-A*, and *GAPDH* in gut tissues (Figure 2E). Similarly, WB tissue showed that *RPS3*, β*-Tubulin, HSP83*, and *eEF2* genes were highly expressed and the most stable genes within these tissues (Table 4), while *RPL17, UbiQ*, and *ArgK* genes were found to be least stably expressed (Figure 2F). The best reference gene combination and stability ranking for WB tissues were *GAPDH, RPS3*, and *Myosin L*. The most stable reference gene among all four tissue stages was *eEF2* after assessing all expression results. The optimal reference genes for all tissue stages combined (head, fat body, gut, and WB) conditions were β*-Tubulin, eEF2*, and *RPS3* based on all algorithms (Supplementary Table 3).

**Table 4:** Ranking of the candidate reference genes based on stability values performed by Delta Ct, BestKeeper, RefFinder, and NormFinder in various tissue types [head, midgut, fat body, and whole body (WB) except head, gut, and fat body (Abdomen)].

**Conditions**	**Genes**	**ΔCt method**		**Best keeper**		**RefFinder**		**NormFinder**		**Recommended genes**
		**Stability**	**Rank**	**Stability**	**Rank**	**Stability**	**Rank**	**Stability**	**Rank**	
Head	*eEF2*	1.21	1	0.6	4	2	1	0.302	7	*RPS3, ArgK* and *eEF2*
	*ArgK*	1.33	2	0.72	6	2.91	3	0.266	4	
	*V-ATPase-A*	1.39	3	0.55	3	3.41	5	0.314	9	
	*RPS3*	1.41	4	0.3	1	2.11	2	0.191	1	
	*β-Tubulin*	1.49	5	1.52	8	5.79	6	0.314	10	
	*UbiQ*	1.5	6	0.92	7	6.24	7	0.266	5	
	*NADH*	1.54	7	0.52	2	3.15	4	0.306	8	
	*Myosin L*	1.63	8	1.71	10	8.46	8	0.257	3	
	*Actin*	1.7	9	1.71	11	9.72	11	0.371	12	
	*HSP83*	1.71	10	1.71	9	9.72	10	0.283	6	
	*GAPDH*	1.71	11	1.81	12	10.98	12	0.232	2	
	*RPL17*	1.74	12	0.68	5	9.64	9	0.335	11	
Fat body	*NADH*	0.97	1	1.18	6	2.21	1	0.363	9	*V-ATPase-A, eEF2* and *NADH*
	*eEF2*	1	2	1.3	7	2.55	3	0.245	4	
	*V-ATPase-A*	1	3	0.73	2	2.45	2	0.250	5	
	*β-Tubulin*	1.07	4	1.13	5	2.99	4	0.332	8	
	*GAPDH*	1.09	5	1.54	10	6.22	7	0.181	1	
	*RPS3*	1.11	6	0.99	4	5.63	6	0.294	6	
	*UbiQ*	1.13	7	0.72	1	3.96	5	0.230	3	
	*HSP83*	1.16	8	1.47	9	8.24	9	0.227	2	
	*ArgK*	1.3	9	0.98	3	7.56	8	0.315	7	
	*Actin*	1.31	10	1.71	11	9.97	10	0.606	11	
	*Myosin L*	1.32	11	1.95	12	10.44	11	0.435	10	
	*RPL17*	1.39	12	1.39	8	10.84	12	0.660	12	
Gut	*eEF2*	1.18	1	1.54	8	3.13	3	0.117	1	*V-ATPase-A, eEF2* and *RPL17*
	*V-ATPase-A*	1.18	2	1.15	7	1.93	1	0.130	2	
	*NADH*	1.24	3	1.09	6	3.57	4	0.270	7	
	*RPL17*	1.26	4	1.05	5	2.99	2	0.262	6	
	*β-Tubulin*	1.48	5	2.21	9	6.51	8	0.215	3	
	*ArgK*	1.54	6	0.7	1	3.81	5	0.396	10	
	*RPS3*	1.54	7	0.81	2	4.6	6	0.283	9	
	*GAPDH*	1.56	8	2.32	10	8.11	9	0.234	4	
	*UbiQ*	1.7	9	0.93	3	6.42	7	0.339	5	
	*Myosin L*	1.93	10	2.84	11	10.49	11	0.279	8	
	*Actin*	1.97	11	2.92	12	11.49	12	0.459	12	
	*HSP83*	1.99	12	0.95	4	8.71	10	0.440	11	
WB	*RPS3*	1.14	1	2.06	6	2.34	2	0.192	3	*RPS3, β-Tubulin* and *HSP83*
	*Actin*	1.18	2	2.16	7	2.3	1	0.375	12	
	*eEF2*	1.19	3	2.27	8	2.91	3	0.247	8	
	*GAPDH*	1.21	4	2.63	9	4.9	5	0.167	2	
	*HSP83*	1.23	5	2.04	5	4.4	4	0.201	4	
	*β-Tubulin*	1.24	6	2	3	5.05	6	0.079	1	
	*V-ATPase-A*	1.57	7	3.04	11	8.1	10	0.273	10	
	*NADH*	1.59	8	1.72	2	5.63	7	0.325	11	
	*Myosin L*	1.62	9	3.08	12	9.39	11	0.233	7	
	*RPL17*	1.75	10	2.01	4	7.95	9	0.215	6	
	*UbiQ*	1.81	11	1.36	1	6.04	8	0.259	9	
	*ArgK*	1.93	12	2.95	10	11.47	12	0.206	5	

#### Identification of Candidate Reference Genes Under Abiotic Conditions

**Figure 3:**
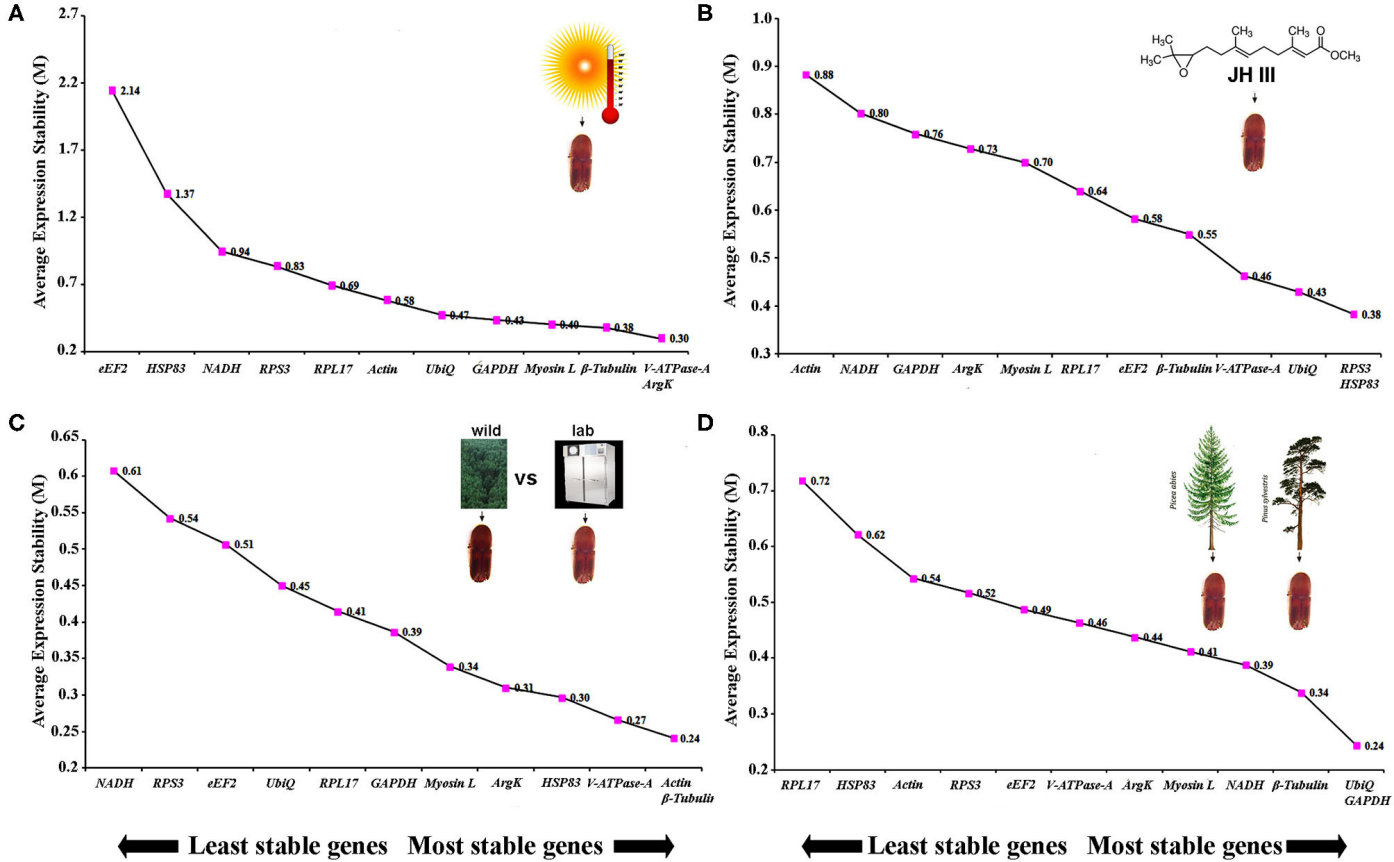
The average expression stability values (M) of 12 reference genes under diverse conditions were calculated by geNorm from the least stable (left) to the most stable (right). **(A)** Temperature, **(B)** juvenile hormone III, **(C)** laboratory-reared vs. wild beetles, and **(D)** pine-fed vs. spruce-fed beetles.

The same four algorithms were used to identify the most appropriate reference genes under four different abiotic conditions such as temperature, JHIII treatment, laboratory-reared vs. wild beetles, and pine-fed vs. spruce-fed beetles. For the temperature treatment, β*-Tubulin, V-ATPase-A, ArgK*, and *GAPDH* genes were shown to have the most stable expression using the four algorithms (Table 5). RefFinder confirmed that the three most stable gene expressions were *V-ATPase-A, ArgK*, and β*-Tubulin* (Figure 3A). After JHIII treatment, the stability of the first four most stable genes, such as *UbiQ, V-ATPase-A, RPS3*, and *Myosin L* determined by four programs was inconsistent (Table 5). The least stable genes were found to be *Actin, RPL17*, and *NADH*. According to RefFinder, the stability ranking of the reference genes of the most stable and the top three candidates were *RPS3, HSP83*, and *UbiQ* (Figure 3B). However, in beetles from wild and laboratory rearing conditions, most stable expressions were observed for *Actin, ArgK, Myosin L*, and *GAPDH via* ΔCt method (Table 5). The top three most stable reference genes *via* RefFinder were *Actin*, β*-Tubulin*, and *V-ATPase-A* (Figure 3C). Similarly, pine- and spruce-fed beetle gut showed stable expression for *V-ATPase-A, UbiQ, ArgK*, and β*-Tubulin* in RefFinder (Table 5 and Figure 3D). The overall stability ranking and combination of reference genes among various tested abiotic conditions were *UbiQ, GAPDH*, and β*-Tubulin*.

**Table 5:** Ranking of the candidate reference genes based on stability values performed by Delta Ct, BestKeeper, RefFinder, and NormFinder under the influence of various abiotic factors such as temperature (Temp), Juvenile hormone III (JHIII), laboratory-reared vs. wild beetles, and pine-fed vs. spruce-fed beetles.

**Conditions**	**Genes**	**ΔCt method**		**Best keeper**		**RefFinder**		**NormFinder**		**Recommended genes**
		**Stability**	**Rank**	**Stability**	**Rank**	**Stability**	**Rank**	**Stability**	**Rank**	
Temp	*β-Tubulin*	0.98	1	1.09	5	1.5	1	0.322	4	*β-Tubulin, V-ATPase-A* and *ArgK*
	*V-ATPase-A*	1.02	2	0.99	4	2	2	0.315	3	
	*ArgK*	1.06	3	1.37	7	3.98	4	0.252	2	
	*GAPDH*	1.07	4	0.93	3	3.66	3	0.356	5	
	*Myosin L*	1.08	5	1.61	9	5.73	6	0.251	1	
	*Actin*	1.13	6	1.29	6	5.73	5	0.519	7	
	*eEF2*	1.17	7	1.5	8	7.24	9	2.051	12	
	*UbiQ*	1.19	8	1.69	10	8.46	10	0.441	6	
	*RPS3*	1.43	9	0.88	2	6.18	8	0.614	8	
	*NADH*	1.59	10	1.79	11	10.24	11	0.668	10	
	*RPL17*	1.94	11	0.86	1	6.04	7	0.630	9	
	*HSP83*	2.6	12	2.73	12	12	12	1.446	11	
JHIII	*V-ATPase-A*	0.85	1	0.54	3	2.34	2	0.225	2	*UbiQ, V-ATPase-A* and *RPS3*
	*UbiQ*	0.87	2	0.45	2	1.86	1	0.208	1	
	*Myosin L*	0.9	3	0.58	4	2.45	3	0.432	10	
	*ArgK*	0.92	4	0.65	5	3.16	5	0.428	9	
	*β-Tubulin*	0.93	5	0.75	8	6.16	6	0.294	3	
	*RPS3*	0.93	6	0.37	1	3.13	4	0.297	4	
	*eEF2*	0.97	7	0.69	7	7.24	7	0.376	8	
	*GAPDH*	1.03	8	0.87	10	8.18	8	0.350	7	
	*HSP83*	1.06	9	0.89	11	9.72	10	0.314	5	
	*Actin*	1.08	10	0.69	6	8.78	9	0.510	12	
	*RPL17*	1.11	11	0.84	9	10.22	11	0.336	6	
	*NADH*	2.55	12	1.78	12	12	12	0.436	11	
Lab vs. Wild	*Actin*	0.9	1	1.68	9	3	2	0.068	2	*Actin, Myosin L* and *ArgK*
	*Myosin L*	0.9	2	1.26	5	2.66	1	0.088	6	
	*ArgK*	0.95	3	1.09	4	3.46	4	0.086	4	
	*GAPDH*	0.95	4	1.65	8	3.36	3	0.103	7	
	*UbiQ*	1.03	5	1.64	7	3.64	5	0.182	9	
	*β-Tubulin*	1.05	6	2.03	10	6.4	6	0.042	1	
	*RPS3*	1.06	7	1.44	6	6.48	8	0.221	10	
	*V-ATPase-A*	1.18	8	0.71	3	6.62	9	0.085	5	
	*HSP83*	1.23	9	2.28	11	9.19	11	0.072	3	
	*eEF2*	1.49	10	2.45	12	10.44	12	0.219	11	
	*RPL17*	1.49	11	0.48	2	7.01	10	0.124	8	
	*NADH*	1.98	12	0.47	1	6.45	7	0.282	12	
Pine vs. Spruce	*UbiQ*	0.65	1	0.52	4	2	2	0.102	2	*V-ATPase-A, UbiQ* and *ArgK*
	*V-ATPase-A*	0.71	2	0.29	1	1.86	1	0.155	4	
	*GAPDH*	0.73	3	0.61	8	4.68	5	0.072	1	
	*ArgK*	0.75	4	0.31	2	2.21	3	0.193	7	
	*β-Tubulin*	0.75	5	0.5	3	2.94	4	0.157	5	
	*Myosin L*	0.79	6	0.59	6	6	6	0.179	6	
	*RPS3*	0.82	7	0.52	5	6.85	7	0.202	8	
	*eEF2*	0.82	8	0.6	7	7.74	8	0.220	10	
	*NADH*	0.86	9	0.8	11	8.89	9	0.149	3	
	*Actin*	1.05	10	0.77	10	10	10	0.212	9	
	*RPL17*	1.3	11	0.74	9	10.46	11	0.393	12	
	*HSP83*	1.36	12	0.8	12	12	12	0.327	11	

### Determination of the Minimum Number of Reference Genes for Normalization

**Figure 4:**
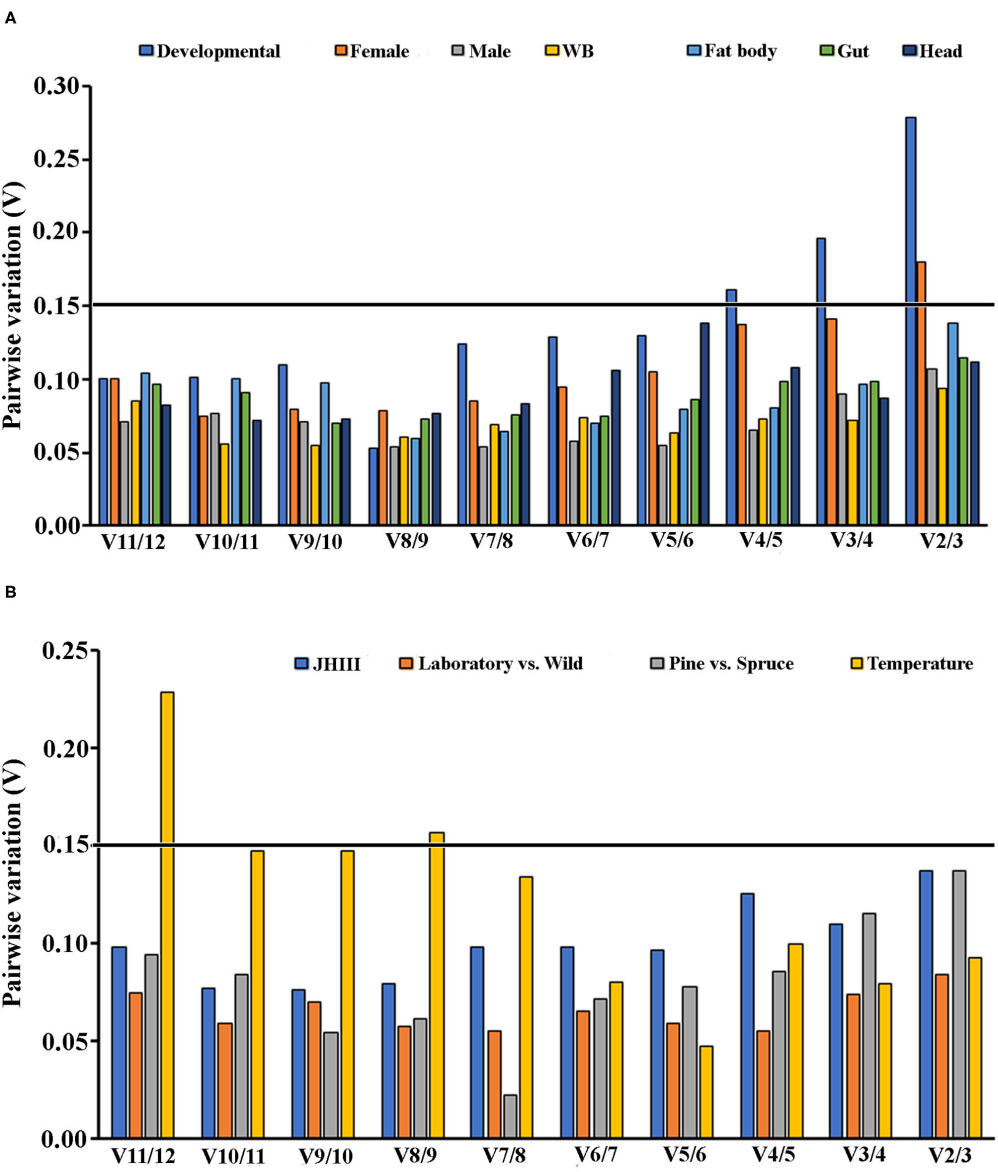
An optimal number of reference genes for the normalization of ISx under selected extrinsic experimental conditions. Based on geNorm analysis, average pairwise variations were calculated between the normalization factors NFn and NFn + 1. Values <0.15 indicate that n + 1 genes were not required for the normalization of gene expression. **(A)** Biotic conditions: developmental stages (i.e., larvae, pupae, and adult stages), tissue types [head, midgut, fat body, and whole body (WB) except head, gut, fat body] and **(B)** Abiotic conditions: different treatments (temperature; Juvenile hormone III; wild-collected vs. long-term laboratory-reared; and different host feeding). Values <0.15 indicate that additional genes are not necessary for normalization.

To generate more accurate and reliable gene expression results, often more than one reference gene is recommended. According to Vandesompele et al. (2002), a Vn/Vn+1 value under 0.15 means adding the n+1 reference gene is unnecessary. Alternatively, the first reference gene is sufficient to normalize the target gene expression in those cases. We also calculated the optimal reference gene number based on geNorm algorithm analysis for each condition. We found that at least two reference genes were required for the head, female tissues, and temperature conditions based on the pairwise values (Figures 4A,B).

### Validation of Reference Gene Selection

**Figure 5:**
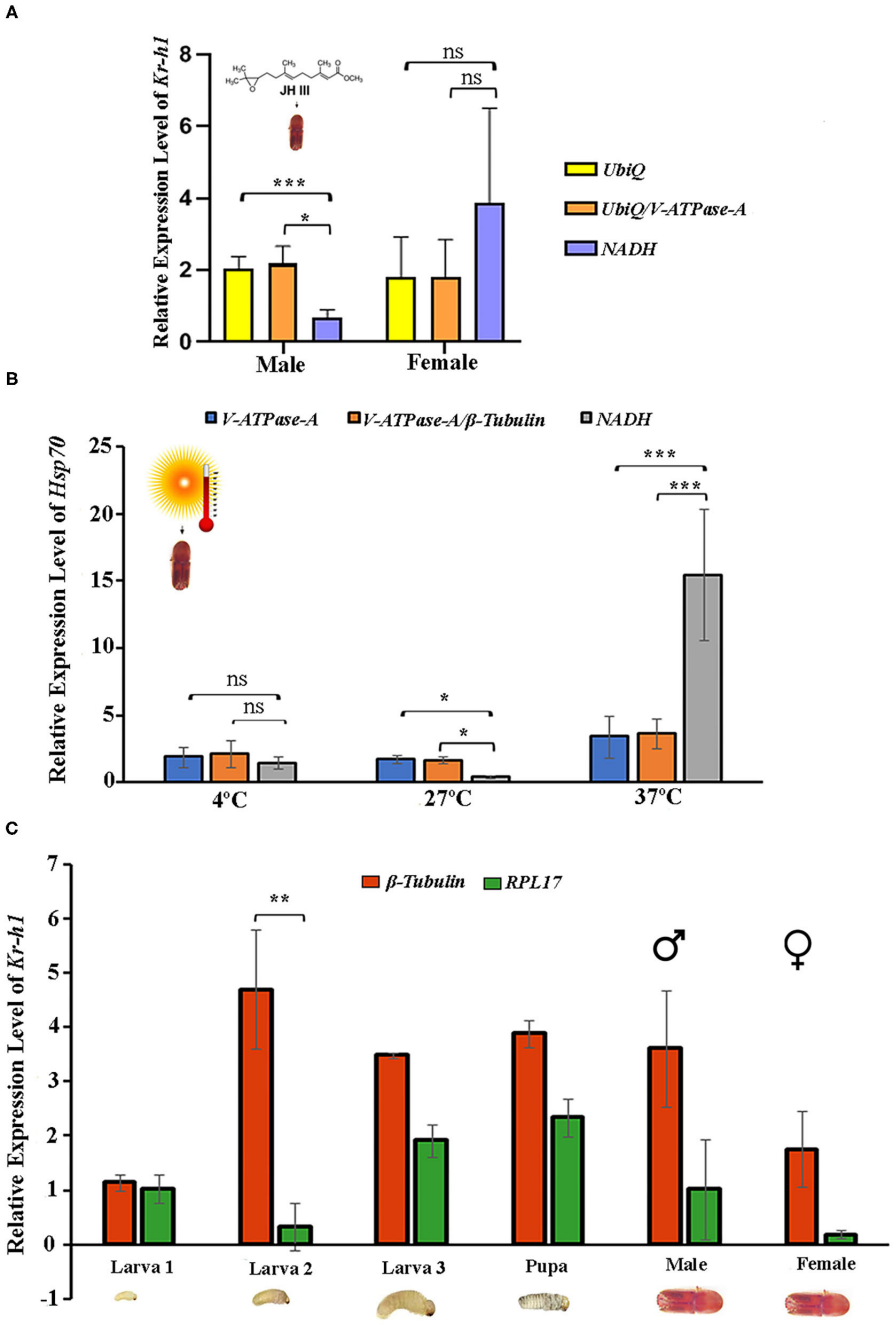
Validation of the recommended reference genes. The relative expression levels of the target genes *Kr-h1* and *Hsp70* were studied under Juvenile hormone III treatment and temperature treatment by normalizing the selected reference gene. The most stable reference gene is *UbiQ* and V-*ATPase-A* for juvenile hormone III; *V*-*ATPase*-*A* and β*-Tubulin* for temperature, the least stable reference gene for both treatments *NADH*. The respective combination of stable reference genes UbiQ+V-*ATPase-A* and *V*-*ATPase*-*A*+β*-Tubulin*. **(A)** Juvenile hormone III and **(B)** Temperature. **(C)** The relative expression levels of the target gene *Kr-h1* were calculated under developmental stages (larvae, pupae, and adult stages), normalized with the most stable reference gene β*-Tubulin* and the least stable reference gene *RPL17*. Data represent mean values ± SD of four biological replicates. Asterisks indicate significant differences in the expression of the target gene normalized separately by different reference genes (***P < 0.001, **P < 0.01, *P < 0.05, ns indicate no significant difference).

*Krüppel-homolog 1* (*Kr-h1*) encodes a key transcription factor and plays a critical role in regulating insect metamorphosis within the juvenile hormone signaling pathway (Li et al., 2018a; Roy and Palli, 2018). The relative expression of *Kr-h1* in response to the JHIII treatment was normalized with single reference genes or gene combinations recommended by geNorm (Figures 4A,B). The two most stable reference genes, individually and in combination, and the least stable gene were used in this experiment. The results showed that the *Kr-h1* gene was expressed in male and female beetles (Figure 5A). The expression levels of *Kr-h1* normalized with *NADH* (least stable) in males reduced from 2.1 to 0.6 fold, and females were increased from 1.8 to 3.8 fold higher than those of *Kr-h1* normalized with stable reference gene or gene combinations, respectively. Similarly, *HSP70* is a key protein closely related to the molecular mechanism underlying insect resistance to the environment (Štětina et al., 2015). The relative expression of *Hsp70* showed a stable expression difference after normalizing with the single and most stable reference gene combination during temperature exposure. On the contrary, the *Hsp70* gene expression level after normalizing with the least stable reference gene showed higher variation after different temperature incubation (Figure 5B). Henceforth, the most stable genes used for normalization, either individually or in combination, resulted in more consistent and trustworthy target gene expression patterns.

In addition, *Kr-h1* gene expression was evaluated for the various developmental stages to validate the reference gene findings taking the most and least stable gene for expression normalization. Results showed that the expression level of *Kr-h1* in the second instar larvae was almost 4-fold higher than the first instar larvae when normalization was performed based on the most stable reference gene (β-*Tubulin*) (Figure 5C). In contrast, when the least stable reference gene (*RPL17*) was used for normalization, the *Kr-h1* expression was considerably low. Furthermore, normalization with β-*Tubulin* resulted in lower expression in all stages except second instar larvae signifying the importance of having optimal reference genes for expression normalization.

## Discussions

The wood-boring coleopteran pest, ISx, is one of the most destructive forest pests, causing severe damage to coniferous species throughout Europe and Asia (Jeger et al., 2017). Environmental stress significantly affects host colonization and can provoke transitions from endemic to epidemic bark beetle development (Kausrud et al., 2012). However, to understand the molecular mechanisms of beetle-host interactions, the advent of high-throughput sequencing technologies must advance to increase genetic information. Reference genes with high expression stability under different environmental conditions will be needed to study further a particular gene expression (Fu and Meyer-Rochow, 2021). However, there is no universal reference gene for all samples and tissue types with diverse conditions to date. Therefore, evaluating the stable reference gene under various treatments is essential before aiming DGE study (Lu et al., 2018). The present study examined 12 reference genes commonly applied to Coleoptera using four algorithms (geNorm, NormFinder, BestKeeper, and the ΔCt method) under various biotic and abiotic conditions. There are copious studies regarding the validation of reference genes in other insects (Lu et al., 2018), but no information has previously been reported in any *Ips* species. Hence, this is the first report for suitable reference genes in any *Ips* bark beetles.

Accurate normalization using a stable reference gene is necessary to conduct gene expression studies under specific experimental conditions and to avoid erroneous differences in target gene expression (Andersen et al., 2004; Bustin et al., 2005; Ferguson et al., 2010; Cheng et al., 2013; García-Reina et al., 2018; Xie et al., 2021). The results obtained in this study indicate that the stability of reference genes in ISx can differ under various experimental conditions, including developmental stage, sex, and tissue-specific conditions, and exposure to abiotic conditions (Tables 2–5 and Figures 1B–4) as observed in other reference gene finding studies in insects (Lu et al., 2018). Among the 12 reference genes studied in this study, we found that β*-Tubulin, eEF2, RPS3*, and *V-ATPase-A* were the most stable in the developmental stage, sex-specific, and tissue-specific conditions from all four algorithms (Tables 2–4); and geNorm (Figures 1B, 2A–F). In addition, both β*-Tubulin* and *eEF2* were more stable than *V-ATPase-A* and *RPS3* at various developmental stages. Teng et al. (2012) reported EF (*eEF2*) as the most stably expressed gene in different developmental stages of *Plutella xylostella*. The higher stability of β*-Tubulin* and *eEF2* in two biotic factors (developmental stages and tissues) was also documented in reference gene analyses for *Agrilus planipennis* (Rajarapu et al., 2012); *Sogatella furcifera* (An et al., 2016); *Mythimna separata* (Li et al., 2018b); *Chilo partellus* (Adeyinka et al., 2019); and *Hippodamia variegate* (Xie et al., 2021). Alternatively, many studies have reported these genes as unsuitable for normalization because of expression variability in different experimental conditions (developmental stages, tissue stages) as in *Drosophila melanogaster* (Ponton et al., 2011). However, our results confirmed that tissue-specific expression of reference gene *eEF2* was highly stable. It was consistently the top-ranking gene for the developmental, head, fat body, and gut except for WB tissue in ISx.

Similarly, RPs have been evaluated and showed highly stable expression in different insects (Lu et al., 2018). Earlier research findings documented that RP-encoding genes are among the most stably expressed reference genes and have been widely used to normalize gene expression levels in insect molecular investigations during the past 10 years (Lu et al., 2018). For instance, *RPS13* and *RPS7* exhibited the most stable expression under larval-crowding conditions in *Mythimna separata* (Li et al., 2018b). *RPS3* also exhibited high stability under larval tissues in *Lucilia sericata* (Baumann et al., 2015). Similarly, *RPL9* and *RPL10* genes showed higher expression stability in different developmental stages and tissues of *Sogatella furcifera* (An et al., 2016), whereas *RPS26* and *RPL32* genes showed the same in *Thermobia domestica* (Bai et al., 2021). Furthermore, *RPS18* and *RPL13* genes showed the highest expression stability in *Rhopalosiphum padi* tissues (Li et al., 2021). Wang et al. (2014) reported *RPL22e* as the most stable reference gene comparing male and female *Mylabris cichorii*. Similarly, our results suggested that the RP, *RPS3*, was the most stable gene among the 12 candidates in sex-specific and tissue-specific (except fat body and gut) conditions tested in ISx (Tables 3, 4 and Figure 2).

Vacuolar-type ATPase (*V-ATPase-A*) is a proton translocating pump responsible for ATP hydrolysis, one of the most highly conserved eukaryotic proteins. The *V-ATPase-A* gene had been commonly used as a reference gene in *Amrasca biguttula* for experiments involving starvation stress and different life stages (Singh et al., 2018); for sex-specific experiments in *Cicindela campestris* (García-Reina et al., 2018). On the contrary, *V-ATPase-A* gene expression was highly unstable in the developmental and tissue stages of *Mythimna separata* (Li et al., 2018c). Nevertheless, our results demonstrated stable expression of *V-ATPase-A* in the fat body and gut tissues of ISx (Table 4 and Figures 2D,E).

Experiments on ISx involving alteration of abiotic conditions revealed a varied set of genes as references, such as *V-ATPase-A, ArgK*, and β*-Tubulin* for temperature incubation; *UbiQ, V-ATPase-A*, and *RPS3* for JHIII treatment; *Actin, Myosin L*, and β*-Tubulin* in between laboratory vs. wild beetles, and *V-ATPase-A, UbiQ*, and *ArgK* among pine vs. spruce-fed beetle gut tissues (Table 5 and Figure 3). The reference gene *V-ATPase-A* was used previously for normalization of *Mythimna separata* gene expression after temperature treatment (Li et al., 2018c), whereas β*-Tubulin* was applied for a similar purpose for *Bemisia tabaci* (Dai et al., 2017); *Phenacoccus solenopsis* (Arya et al., 2017); *Amphitetranychus viennensis* (Yang et al., 2019); and *Hippodamia variegate* (Xie et al., 2021).

In the present study, host plants among all treatments caused the highest expression variations of the reference genes. In the dataset of gut samples collected from the adults fed on different plants (i.e., pine and spruce), the stability ranking of tested reference genes was different according to five algorithms. The most stable reference gene was *UbiQ* and *GAPDH* according to the geNorm algorithm (Figure 3D). However, *V-ATPase-A, UbiQ*, and *ArgK* were the most stable reference genes evaluated by the other four algorithms (delta Ct, BestKeeper, RefFinder, and Normfinder, respectively) (Table 5). Interestingly, similar studies in other arthropods have not found such dramatic variations so far (Arya et al., 2017). Furthermore, in laboratory-reared and wild-collected beetle gut tissues, *Actin, Myosin L*, and *ArgK* were the most stable reference genes after delta Ct, BestKeeper, RefFinder, and Normfinder analysis (Table 5), which differed from the geNorm algorithm analysis (Figure 3C). In our opinion, plant diet causes drastic changes at the gene expression level in ISx, hence higher variation in the reference gene expression.

Further, the expression of *Kr-h1* and *Hsp70* was evaluated in various developmental stages and respective treatments (JHIII and temperature) to corroborate the suitability of the identified reference genes. Although, the results demonstrated that the expression trends in different conditions were accordant using various reference gene or gene combinations. The *Kr-h1* gene expression was induced by JHIII, as expected from similar treatments on other beetles (Roy et al., 2017; Roy and Palli, 2018; Xu et al., 2018). However, our results displayed that using less stable reference genes may generate erroneous interpretation, whereas stable reference gene combinations can reduce bias during normalization (Figure 5A). Alternatively, there was no significant expression difference of *Hsp70* at 4°C temperature, but considerable expression differences were observed after 27 and 37°C incubation using the most stable reference gene (β*-Tubulin*) separately and together (β*-Tubulin/ V-ATPase-A*), and least reference gene (*NADH*) as normalizer. It is worth mentioning that the expression change was more extensive at 37°C for *Hsp70* when normalized with *NADH*, the least stable gene. Recently, elimination of such erroneous findings was achieved by normalization of gene expression with combinations of stable reference genes in different experimental conditions in *Helicoverpa armigera* (Zhang et al., 2015), *Aphis gossypii* (Ma et al., 2016), and *Cydia pomonella* (Wei et al., 2020). Additionally, with the selected single reference genes, such as β*-Tubulin* (most stable) and *RPL17* (least stable), we performed RT-qPCR to study *Kr-h1* expression patterns in several developmental stages of ISx (Figure 5C). More convincing results were obtained when two genes were used for expression normalization (Figures 5A,B). Our results corroborate the current notion of using two or three genes for target gene expression normalization to enhance accuracy (Arya et al., 2017; Li et al., 2018c; Wei et al., 2020; Bai et al., 2021; Fu and Meyer-Rochow, 2021).

Similar to other published studies in the field, our analyses also documented different results under diverse experimental conditions for all five algorithms; however, the results were comparable among some treatments. Interestingly, the most conclusive observation from the current assessments showed that β*-Tubulin* and *eEF2* were the most stable reference genes across all developmental stages, sex, and tissue-specific conditions investigated under the present study (Tables 2–4 and Figures 1A, 2). Our findings suggested that the recommended number of reference genes should be two for many comparisons in the study, based on the pairwise values (>0.15) obtained with geNorm (Figures 4A,B). Furthermore, these results imply that a single reference gene is not optimal to normalize the target gene expression in different experimental conditions. Hence, we endorsed optimal reference genes for the specific experimental conditions in ISx (Table 6).

**Table 6:** Summary of the recommended reference genes under different experimental conditions.

**Conditions**	**Recommendation**
**Biotic conditions**	
Developmental stage	*β-Tubulin, eEF2*, and *RPS3*
**Sex-specific tissues**	
Male	*RPS3, GAPDH*, and *eEF2*
Female	*eEF2, RPS3*, and *β-Tubulin*
**Tissue**	
Head	*RPS3, ArgK*, and *eEF2*
Fat body	*V-ATPase-A, eEF2*, and *NADH*
Gut	*V-ATPase-A, eEF2*, and *RPL17*
Abdomen (WB)	*RPS3, β-Tubulin*, and *HSP83*
Between tissues	*β-Tubulin, RPS3, and eEF2*
**Abiotic conditions**	
Temperature	*β-Tubulin, V-ATPase-A*, and *ArgK*
Juvenile hormone III	*UbiQ, V-ATPase-A*, and *RPS3*
Laboratory-reared vs. wild beetles	*Actin, Myosin L*, and *ArgK*
Pine-fed vs. spruce-fed	*V-ATPase-A, UbiQ*, and *ArgK*

In conclusion, temperate and boreal forests have recently undergone unprecedented pressure from bark beetle outbreaks, reducing forest biodiversity and their role in global carbon sequestration. It also affects their economic value and endangered wildlife habitat. Current management approaches are proven progressively deficient against outbreaking bark beetle populations, urging investigations into novel mitigation strategies using state-of-the-art molecular methodologies. Future gene expression and functional genomics studies are crucial to alleviate the ongoing bark beetle-mediated forest depletion. Hence, dedicated reference gene selection studies are of utmost importance on economically important bark beetles. The present work represents the first reference gene validation study in *I. sexdentatus*, an ecologically important wood-boring beetle. It provides valuable information on reference genes (Table 6) for future molecular studies (i.e., DGE studies) on host-beetle interactions and functional genomic studies (i.e., RNAi) on this bark beetle and similar *Ips* beetles (Coleoptera: Curculionidae: Scolytinae).

## Data Availability Statement

The original contributions presented in the study are included in the article/Supplementary Material, further inquiries can be directed to the corresponding author/s. The reference gene sequences used in the study were submitted under the NCBI accession numbers from OK143445 to OK143455.

## Author Contributions

GS, AC, and AR conceived and designed the research and prepared the final manuscript. SA, RM, and JS collected, sorted, and dissected beetles. JB and SA prepared samples for in-house transcriptome. GS conducted real-time experiments and wrote the first draft. GS and MKS analyzed the data. All authors have read and approved the final manuscript.

## Funding

The project was funded by the Internal Grant Agency (IGA) from the Faculty of Forestry and Wood Sciences, Czech University of Life sciences. Infrastructural support and salary for GS, JB, and AR are obtained from grant EXTEMIT-K, No. CZ.02.1.01/0.0/0.0/15_003/0000433 financed by OP RDE. The financial support for AC was provided by grant EVA 4.0, No. CZ.02.1.01/0.0 /0.0/16_019 /0000803 financed by OP RDE.

## Conflict of Interest

The authors declare that the research was conducted in the absence of any commercial or financial relationships that could be construed as a potential conflict of interest.

## Publisher's Note

All claims expressed in this article are solely those of the authors and do not necessarily represent those of their affiliated organizations, or those of the publisher, the editors and the reviewers. Any product that may be evaluated in this article, or claim that may be made by its manufacturer, is not guaranteed or endorsed by the publisher.
