# Supplementary material for: Reference Gene Selection for Normalizing Gene Expression in Ips Sexdentatus (Coleoptera: Curculionidae: Scolytinae) Under Different Experimental Conditions
Source: Front Physiol. 2021 Oct 27;12:752768. doi: 10.3389/fphys.2021.752768 (PMC8580292; doi:10.3389/fphys.2021.752768)
Supplement: Supplementary file 1 [file Table_1.DOCX]

**Supplementary Table S1**. Summary of the reference gene studies in insects from Coleoptera 2010 to 2020

| **Gene** | **Genus Name** | **Reference** |
| --- | --- | --- |
| *Actin* | *Ips typographus* | unpublished |
|  | *Harmonia axyridis* | Yang et al., 2018 |
|  | *Agrilus planipennis* | Rajarapu et al., 2012 |
|  | *Diabrotica undecimpunctata howardi* | Basu et al., 2019 |
|  | *Diabrotica virgifera virgifera* | Rodrigues et al., 2013 |
|  | *Galeruca daurica* | Tan et al., 2017 |
|  | *Hippodamia convergens* | Pan et al., 2015 |
|  | *Coccinella septempunctata* | Yang et al., 2016 |
|  | *Coleomegilla maculata* | Yang et al., 2015 |
|  | *Cryptolestes ferrugineus* | Tang et al., 2017 |
|  | *Mylabris cichorii* | Wang et al., 2014; |
|  | *Tribolium castaneum* | Lord et al., 2010; Toutges et al., 2010; Sang et al., 2015 |
|  | *Anthonomus eugenii* | Pinheiro and Siegfried 2020 |
|  | *Propylea japonica* | Lu et al., 2018 |
|  | *Leptinotarsa decemlineata* | Shi et al., 2013 |
|  | *Colaphellus bowringi* | Tan et al., 2015 |
|  | *Dendroctonus valens* | Zheng et al., 2020 |
| *eEF* | *Agrilus planipennis* | Rajarapu et al., 2012 |
|  | *Anoplophora glabripennis* | Rodrigues et al., 2017 |
|  | *Leptinotarsa decemlineata* | Shi et al., 2013 |
|  | *Diabrotica virgifera virgifera* | Rodrigues et al., 2013 |
|  | *Colaphellus bowringi* | Tan et al., 2015 |
|  | *Hippodamia convergens* | Pan et al., 2015 |
|  | *Coccinella septempunctata* | Yang et al., 2016 |
|  | *Coleomegilla maculata* | Yang et al., 2015 |
|  | *Ips typographus* | unpublished |
|  | *Cryptolestes ferrugineus* | Tang et al., 2017 |
|  | *Mylabris cichorii* | Wang Y. et al., 2014 |
|  | *Diabrotica undecimpunctata howardi* | Basu et al., 2019 |
|  | *Anthonomus eugenii* | Pinheiro and Siegfried 2020 |
|  | *Propylea japonica* | Lu et al., 2018 |
| *Β-Tubulin* | *Cicindela campestris; C. littoralis* | García-Reina et al., 2018 |
|  | *Ipstypographus* | unpublished |
|  | *Anoplophora glabripennis* | Rodrigues et al., 2017 |
|  | *Diabrotica undecimpunctata howardi* | Basu et al., 2019 |
|  | *Agrilus planipennis* | Rajarapu et al., 2012 |
|  | *Diabrotica virgifera virgifera* | Rodrigues et al., 2013 |
|  | *Galeruca daurica* | Tan et al., 2017 |
|  | *Colaphellus bowringi* | Tan et al., 2015 |
|  | *Mylabris cichorii* | Wang Y. et al., 2014 |
|  | *Tribolium castaneum* | Sang et al., 2015 |
|  | *Propylea japonica* | Lu et al., 2018 |
|  | *Dendroctonus valens* | Zheng et al., 2020 |
| *Myosin L* | *Cicindela campestris; C. littoralis* | García-Reina et al., 2018 |
|  | *Ips typographus* | unpublished |
| *V-ATPase* | *Cicindela campestris; C. littoralis* | García-Reina et al., 2018 |
|  | *Ips typographus* | unpublished |
|  | *Coleomegilla maculata* | Yang et al., 2015 |
|  | *Hippodamia convergens* | Pan et al., 2015 |
|  | *Propylea japonica* | Lu et al., 2018 |
|  | *Dendroctonus valens* | Zheng et al., 2020 |
| *NADH* | *Ips typographus* | unpublished |
|  | *Cicindela campestris; C. littoralis* | García-Reina et al., 2018 |
|  | *Coccinella septempunctata* | Yang et al., 2016 |
|  | *Coleomegilla maculata* | Yang et al., 2015 |
|  | *Ips typographus* | unpublished |
| *UbiQ* | *Mylabris cichorii* | Wang Y. et al., 2014 |
|  | *Diabrotica undecimpunctata howardi* | Basu et al., 2019 |
|  | *Ips typographus* | unpublished |
|  | *Agrilus planipennis* | Rajarapu et al., 2012 |
|  | *Anoplophora glabripennis* | Rodrigues et al., 2017 |
|  | *Harmonia axyridis* | Yang et al., 2018 |
| *GAPDH* | *Ips typographus* | unpublished |
|  | *Agrilus planipennis* | Rajarapu et al., 2012 |
|  | *Anoplophora glabripennis* | Rodrigues et al., 2017 |
|  | *Diabrotica undecimpunctata howardi* | Basu et al., 2019 |
|  | *Diabrotica virgifera virgifera* | Rodrigues et al., 2013 |
|  | *Galerucadaurica* | Tan et al., 2017 |
|  | *Colaphellus bowringi* | Tan et al., 2015 |
|  | *Harmonia axyridis* | Yang et al., 2018 |
|  | *Hippodamia convergens* | Pan et al., 2015 |
|  | *Coleomegilla maculata* | Yang et al., 2015 |
|  | *Cryptolestes ferrugineus* | Tang et al., 2017 |
|  | *Tribolium castaneum* | Toutges et al., 2010, Sang et al., 2015 |
|  | *Propylea japonica* | Lu et al., 2018 |
|  | *Anthonomus eugenii* | Pinheiro and Siegfried 2020 |
| *ArgK* | *Leptinotarsa decemlineata* | Shi et al., 2013 |
|  | *Coccinella septempunctata* | Yang et al., 2016 |
|  | *Coleomegilla maculata* | Yang et al., 2015 |
|  | *Ips typographus* | unpublished |
|  | *Cicindela campestris; C. littoralis* | García-Reina et al., 2018 |
|  | *Propylea japonica* | Lu et al., 2018 |
|  | *Anthonomus eugenii* | Pinheiro and Siegfried 2020 |
| *RPS3* | *Tribolium castaneum* | Lord et al., 2010; Toutges et al., 2010; Sang et al., 2015 |
| *RPL* | *Coleomegilla maculata* | Yang et al., 2015 |
|  | *Diaphania caesalis* | Wang et al., 2020 |
| *Hsp90* | *Harmonia axyridis* | Yang et al., 2018 |
|  | *Coleomegilla maculata* | Yang et al., 2015 |
|  | *Propylea japonica* | Lu et al., 2018 |

Basu, S., Pereira, A. E., Pinheiro, D. H., Wang, H., Valencia-Jiménez, A., Siegfried, B. D., et al. (2019). Evaluation of reference genes for real-time quantitative PCR analysis in southern corn rootworm, *Diabrotica undecimpunctata howardi* (Barber). *Sci. Rep.* 9:10703. doi.org/10.1038/s41598-019-47020-y

García-Reina, A., Rodríguez-García, M. J., and Galián, J. (2018). Validation of reference genes for quantitative real-time PCR in tiger beetles across sexes, body parts, sexual maturity and immune challenge. *Sci. Rep***.**8, 10743. doi.org/10.1038/s41598-018-28978-7

1. Lord, J. C., Hartzer, K., Toutges, M., and Oppert, B. (2010). Evaluation of quantitative PCR reference genes for gene expression studies in *Tribolium castaneum* after fungal challenge. *J. Microbiol. Methods.* 80(2), 219-21. doi: 10.1016/j.mimet.2009.12.007.

Lu, J., Yang, C., Zhang, Y., and Pan, H. (2018). Selection of Reference Genes for the Normalization of RT-qPCR Data in Gene Expression Studies in Insects: A Systematic Review. *Front. Physiol*. 9:1560. doi:10.3389/fphys.2018.01560

1. Pan, H. P., Yang, X. W., Siegfried, B. D., and Zhou, X. (2015). A comprehensive selection of reference genes for RT-qPCR analysis in a predatory lady beetle, *Hippodamia convergens* (Coleoptera: Coccinellidae). *PLoS ONE,* 10:e0125868. doi: 10.1371/journal.pone.0125868
2. Pinheiro, D.H., Siegfried, B.D. Selection of reference genes for normalization of RT-qPCR data in gene expression studies in Anthonomus eugenii Cano (Coleoptera: Curculionidae). Sci Rep 10, 5070 (2020). <https://doi.org/10.1038/s41598-020-61739-z>
3. Rajarapu, S. P., Mamidala, P., and Mittapalli, O. (2012). Validation of reference genes for gene expression studies in the emerald ash borer (*Agrilus planipennis*). *Insect Sci*. 19, 41–46. doi.org/10.1111/j.1744-7917.2011.01447.x

Rodrigues, T. B., Khajuria, C., Wang, H., Matz, N., Cunha, C. D., Valicente, F. H., et al. (2014). Validation of reference housekeeping genes for gene expression studies in western corn rootworm (*Diabrotica virgifera virgifera*). *PLoS ONE.* 30;9(10):e109825. doi: 10.1371/journal.pone.0109825.

1. Sang, W., He, L., Wang, X.P., Zhu-Salzman, K., and Lei, C. L. (2015). Evaluation of reference genes for RT-qPCR in *Tribolium castaneum* (Coleoptera: Tenebrionidae) under UVB stress. *Environ. Entomol*. 44:418–425. doi.org/10.1093/ee/nvv010 PMID: 26313197.
2. Shi, X. Q., Guo, W. C., Wan, P. J., Zhou, L. T., Ren, X. L., Ahmat, T., Fu, K. Y., and Li, G. Q. (2013). Validation of reference genes for expression analysis by quantitative real-time PCR in *Leptinotarsa decemlineata* (Say). *BMC Res. Notes*, 6, 93. doi.org/10.1186/1756-0500-6-93
3. Tan, Q. Q., Zhu, L., Li, Y., Liu, W., Ma, W. H., Lei, C. L., and Wang, X. P. (2015). A de novo transcriptome and valid reference genes for quantitative real-time PCR in *Colaphellus bowringi*. *PLoS ONE*. 18,10(2):e0118693. doi: 10.1371/journal.pone.0118693.
4. Tan, Y., Zhou, X. R., and Pang, B. P. (2017). Reference gene selection and evaluation for expression analysis using qRT-PCR in *Galeruca daurica* (Joannis). *Bull. Entomol. Res.* 107, 359–368. doi: 10.1017/s0007485316000948
5. Tang, P. A., Duan, J. Y., Wu, H. J. Ju X. R., and Yuan, M. L. (2017). Reference gene selection to determine differences in mitochondrial gene expressions in phosphine-susceptible and phosphine-resistant strains of *Cryptolestes ferrugineus*, using qRT-PCR. *Sci. Rep*. 7, 7047. doi.org/10.1038/s41598-017-07430-2
6. Toutges, M.J., Hartzer, K., Lord, J., and Oppert, B. (2010). Evaluation of reference genes for quantitative polymerase chain reaction across life cycle stages and tissue types of *Tribolium castaneum*. *J. Agric. Food Chem*. 58(16), 8948–8951. /doi.org/10.1021/jf101603j
7. Wang, Y., Wang, Z. K., Huang, Y., Liao, Y. F., and Yin, Y. P. (2014). Identification of suitable reference genes for gene expression studies by qRT-PCR in the blister beetle *Mylabris cichorii*. *J. Insect Sci*. 14:94. doi: 10.1093/jis/14.1.94.
8. Wang Z, Meng Q, Zhu X, Sun S, Liu A, Gao S, et al. (2020). Identification and evaluation of reference genes for normalization of gene expression in developmental stages, sexes, and tissues of *Diaphania caesalis* (Lepidoptera, Pyralidae). *J. Insect Sci*. 20, 1–9.
9. Yang, C. X., Pan, H. P., Noland, J. E., Zhang, D. Y., Zhang, Z. H., Liu, Y., et al. (2015). Selection of reference genes for RT-qPCR analysis in a predatory biological control agent, *Coleomegilla maculata* (Coleoptera: Coccinellidae). *Sci. Rep.* 5:18201. doi: 10.1038/srep18201
10. Yang, C., Preisser, E. L., Zhang, H., Liu, Y., Dai, L., Pan, H., and Zhou, X. (2016). Selection of Reference Genes for RT-qPCR Analysis in *Coccinella septempunctata* to Assess Un-intended Effects of RNAi Transgenic Plants. *Front. Plant Sci.* 7, 1672. doi.org/10.3389/fpls.2016.01672
11. Yang, X., Pan, H., Yuan, L., and Zhou, X. (2018). Reference gene selection for RT-qPCR analysis in *Harmonia axyridis*, a global invasive lady beetle. *Sci. Rep*. 8, 2689. <https://doi.org/10.1038/s41598-018-20612-w>
12. Zheng, C., Zhao, D., Xu, Y., Shi, F., Zong, S., and Tao, J. (2020). Reference Gene Selection for Expression Analyses by qRT-PCR in *Dendroctonus valens*. *Insects.* 27, 11(6):328. doi: 10.3390/insects11060328.
