# Supplementary material for: Reference Gene Selection for Normalizing Gene Expression in Ips Sexdentatus (Coleoptera: Curculionidae: Scolytinae) Under Different Experimental Conditions
Source: Front Physiol. 2021 Oct 27;12:752768. doi: 10.3389/fphys.2021.752768 (PMC8580292; doi:10.3389/fphys.2021.752768)
Supplement: Supplementary file 2 [file Table_2.DOCX]

**Table S2**: List of 12 reference gene sequences used in the present study based on in-house transcriptome of *Ips sexdentatus*.

>β-actin (Actin)

ATCGTAGAAGGAGCCAATGCGGTGATTTCTTTCTGCATACGGTCAGCAATACCAGGGTACATAGTGGTACCTCCAGAGAGGACAGTGTTTGCGTACAGATCCTTCCTGATGTCCACATCACACTTCATGATGGAGTTGTAGACGGTTTCGTGGATACCGCAAGCTTCCATTCCCAAGAAAGATGGCTGGAATAGAGCTTCGGGACAACGGAATCTTTCATTCCCAATAGTAATCACTTGTCCGTCTGGCAATTCATAACTCTTCTCTAGGGAACTAGAACTGGCGGCAGTCGCCATTTCTTGCTCAAAGTCGAGGGCAACGTAGCATAACTTTTCCTTAATGTCACGTACAATTTCTCTTTCGGCAGTGGTGGTGAAAGAATATCCTCGTTCGGTGAGAATTTTCATTAGGTAGTCGGTCAAGTCACGACCGGCTAAATCCAGACGAAGAATAGCGTGAGGAAGGGCATAGCCTTCATAGATTGGAACCGTGTGAGATACTCCGTCGCCCGAATCGAGTACAATACCTGTTGTACGACCGGAAGCGTATAATGATAGAACGGCTTGGATAGCCACGTACATGGCGGGTGTGTTAAAGGTCTCAAACATGATTTGGGTCATTTTCTCCCTGTTAGCTTTAGGGTTCAATGGCGCTTCAGTAAGAAGAACTGGATGTTCTTCAGGAGCCACTCGAAGTTCATTGTAAAACGTGTGATGCCAAATCTTCTCCATGTCATCCCAGTTGGTCACAATACCATGTTCAATTGGGTAC

>translation elongation factor (eEF2)

GTGACGTCGTAGTCGTACTTCTCTCCCAAATAACGAGCCCTAGCTTTGAATTCGTCACGGGGATTGACTTTACCGTCGTCGATGTCTTCAGCCAAACCATCAGGCATGGGTTGAGCTTTCATGAACAATCTGTTGTGCTTGTTGGGAGATTTGGACAGACACATCTGGTCAGATTCTTCGCTAACGGTTTCACGGTAAGACACAACAGGGTCGGATTTCTTGATTGGGATACAAGCGTGGTCGTCTTCCAAATCTTTCAAACAAATCTCCAAGTGGAGTTCTCCGGCACCGGCGATGATGTGTTCACCGGATTCTTCAATGATACATTGGACCATAGGGTCGGATTTAGCGAGACGTTTTAGACCTTCAACAAGTTTGGGCAAATCAGCGGGGTTCTTAGGTTCAACAGCAACACGTACGACGGGAGACACACTGAACTTCATGACCTTCAAGTTATGGGCGTCTTTGAACGTAGTGATGGTACCGGTTTTGACCAAAAACTGATCGACACCGACGAGACCGCAAATGTTTCCAGATGGGACATCCTCGATGGCCTCAACGTAACGACCCATCATCAGAATGGTTCTTTGGATGGCCTTTTCGTACAAATCTTCTTTCTTTCCGGGCACGTAGTTGGGTCCCATAATGCGAGCCTTCATTCCGGTGGCTACTTTACCGGAGAACACACGACCAAAGGCGTAGAAACGACCTTTGTCGGAGGTCGGTACCATTTTTGACACGTACATCATCAAAGGACCGTTGGGATCGCAAGTTTTGATACCAAGAGCAGCTTCATCATCGTGGGGTCCTTCATACAACATCTCCATTCTGTACTTTTGAGCTGTCACAGGTGAAGGCAAATGAATGGCAATCATCTGAAGCAGAGCCTCTCCAGCAGGCAACCATGTACGCATCACAACCTTCAATAACTGTTTTCCGTCCTTGTCCTTGTCTTCATGTTTTAAAGCAATGCCCAATTTTTGCAAAAGTGATTCGCACTCGTCTTTCTTGTAGTTCATGATGGCATCGAAAATTTTGTAGATAGGATCCAAGATGTACATGCAGAATGAACGTTTGTTGTCGGCGTCCTTTTGCTTGGCCCACTTCTTGGTTTTTGGGTTGAAGAAGTTCTCACCCCAGAGACGGTTCATTAGTTTGACGACGTCGATCTTGAATTTTTCAGCGTACATTTCGGCGAATTGTTTCAGAGTGAAAGCCCATCCGTGCAACCCTGATCCGAATCCTACTGAACCTTTACTAGGATCAACGCGCACTTCTCCCATCGGACCGCTGTCATCGTTGTAGGTAGCGATGATGACGTTTACGTTTTCGACGATACGCTGGAACGTTTGGAACAGTTCCTCAGAGCCCAATTGCAATTCGAGCAGAGCACGGTCCATTTTGTTCATGAACAAAATGGGCTTAATACGCTCGGCGATAGCTTGACGCAGTACAGTTTCAGTTTGTACACACACACCTGATACACAGTCCACAACAACAAGAGCACCATCAGTTACACGGAGAGCAGCAGTTACTTCAGATGAAAAGTCCACATGTCCGGGAGAGTCAATCAAATTGATTAAGAAACCTTTTTCTTCCTTTTCACGTTGGTCCGGGCTGGTAATAAAGACAAGATCCTTGGTGTCAAGCTCAAAGAACATAGATATGGCCGTTGATTTGATGGTAATACATCTTTCCTGTTCATCTTTTCTGGTGTCGGTGAATCTTGTTTCTCCAGCTTTAGCACCGGCAATAATACCAGCCTTGGACACTAGGGAGTCTGTAAGGGTGGATTTTCCATGGTCCACGTGGGCAATGACGGACATATTACGGATATTCCGCTTTTTGTCCATCATGACACGGATCTCGTCAACAGTGAAGTTCACCAT

>Tubulin beta-1 chain (β-Tubulin)

TTAGTTCTCGTCAACTTCCTGTTCCTGGTCTTCATCGAATTCGGCATCTTCATCGGCGGTGGCTTCCTGGTACTGTTGGTACTCGGACACCAAATCGTTCATGTTGGATTCAGCTTCGGTGAATTCCATCTCGTCCATACCCTCTCCAGTGTACCAATGCAAGAAAGCCTTACGCCTGAACATGGCGGTGAACTGTTCGGAGATCCTCTTGAACAACTCCTGGATGGCGGTCGAGTTACCGATGAAGGTGGCGGACATTTTCAATCCCCTGGGTGGGATGTCGCACACGGCGGTTTTCACGTTGTTCGGGATCCATTCGACGAAGTAGCTGCTGTTCTTGTTCTGGATGTTGAGCATCTGTTCGTCCACCTCCTTCATGGACATGCGTCCTCTGAAGACGGCGGCGACGGTCAGGTAACGTCCGTGTCGGGGATCGCAGGCGGCCATCATGTTTTTGGCGTCGAACATTTGTTGGGTCAATTCGGGCACGGTGAGGGCGCGGTACTGCTGGCTGCCCCTGGATGTTAAGGGGGCGAAACCGGGCATGAAGAAGTGGAGACGGGGGAACGGTACCATGTTCACTGCTAGTTTACGGAGGTCAGCGTTTAATTGACCTGGGAACCTAAGACACGTCGTAACCCCGGACATTGTCAGTGAAACCAAGTGATTCAAGTCTCCGTAGGTGGGTGTGGTGAGTTTCAGCGTTCTGAAGCAGATGTCGTAGAGAGCTTCGTTATCGATGCAGTAAGTTTCGTCTGTGTTTTCGACCAATTGATGTACGGACAAGGTGGCATTGTAGGGTTCCACGACGGTGTCGGATACTTTGGGTGAGGGGACCACCGAATAAGTGTTCATAATCCTGTCCGGGTATTCCTCCCTGATCTTGGAGATCAACAGGGTGCCCATACCGGAGCCGGTACCGCCGCCCAGCGAGTGGGTCAACTGGAATCCTTGCAAACAGTCGCAGGACTCGGCCTCTTTCCTCACCACGTCCAACACGGAATCGACGAGCTCAGCCCCTTCCGTGTAATGGCCCTTCGCCCAGTTGTTACCGGCTCCGGACTGTCCGAAGACGAAGTTGTCCGGTCTGAAGATCTGTCCGAAGGGGCCGGATCTGACCGAGTCCATGGTGCCGGGCTCCAGGTCGACGAGGATGGCACGGGGCACGTATTTACCGCCTGAGGCTTCATTGTAGTATACGTTGATTCTCTCGAGCTGGAGGTCTGAGTCACCGTGGTATGCTCCGGTGGGGTCGATACCGTGCTCGTCGGAGATGATTTCCCAGAATTTGGCTCCAATTTGGTTACCGCACTGGCCGGCTTGAATGTGTACGATTTCCCTCAT

>myosin regulatory light chain 2 (Myosin L)

GTCAATGGTACCGTTTTCGTCGAACGACCTGTAGGCAGCGGCTACGACCTCATCGTCGTCACTACCACCAGAATCGGCCATACGGGCGCCGAAAAGACCCAACAACTGGGTAAGGTTGATGGGACCTGGGGCTTCGCGCACCTTTTCGTCCAACTCCTTTTCGCTGGCGATTTTGCCGACGGCGTCGAACGTCGCCCTCAAGTCTTCTTTGCTGATGATACCGTCTTTGTCGTGGTCCATTAGGTTGAAGGCTTCCTTGAACTCAGCCACATGAGCCGGGGAGAACATGGAGAACACATTGGAGCCGGAACGTTTGGCCCTCTTCGAGGTGCTCTTGGAGGACGTGCGGGTCTCAGCGGCG

>V-type proton ATPase catalytic subunit A (V-ATPase-A)

GAGAAGGAGTCCACGTATGGATTCGTGTACGGAGTCTCCGGACCTGTCGTAACCGCGGAGAAGATGGCCGGATCCGCTATGTACGAGCTGGTGCGTGTCGGACACGGCGAGCTGGTCGGAGAGATCATCCGTCTTGAGGGTGACTTCGCCACCATCCAAGTATACGAAGAGACTTCCGCTGTGACGATCGGAGATCCCGTGCTCCGTACCGGAAAGCCCCTTTCCGTCGAGCTGGGTCCTGGCATTATGGGTTCAATTTTTGACGGTATCCAGCGTCCCCTGAAGGACATTGCCGACCTCACTCAGTCCATCTACATTCCCAAGGGAGTCAACACAAACGCCCTCTCCCGTGACGCTCGCTGGGACTTTGAGGTGACCAAGGACATCCGCGTGGGATCCCACGTCACCGGCGGAGACATCATCGGACTCGTGCACGAGAACGTTCTCATCAAGCACAGAATCATGATCCCACCCAGTGCGTGCGGAACCGTCACCTTCCTGGCCCCATCTGGACAGTACACCGTGACTGATGTCTTGGTTGAGATCGAGTTTTCCGGCGAGAAGCAGCAGTTTTCTATGCTCCAGATCTGGCCCGTGCGTAGCCCTCGTCCAGTCAGCGAGAAATTGGCGGCCAACAACCCACTTCTCTGCGGTCAGCGTGTGCTTGACGCTCTGTTCCCATGTGTGCAGGGAGGAACCACCGCCATCCCCGGAGCTTTCGGTTGCGGAAAGACTGTGATCTCGCAGTCACTCTCCAAGTACTCCAACTCGGACGGAATTATTTACGTCGGATGCGGAGAGCGCGGAAACGAAATGTCTGAGGTACTTCGTGATTTCCCCGAGCTGACTATGGAGGTCGATGGAGTGACCACTTCTATCATGAAGCGTACCGCTCTAGTCGCTAACACTTCGAACATGCCTGTGGCTGCTCGTGAAGCTTCCATCTACACTGGAATCACTCTCGCCGAGTACTTCCGTGATATGGGTTTGAACGTGGCTATGATGGCCGACTCTACATCTCGATGGGCTGAGGCTCTTCGTGAGATTTCAGGACGTCTGGGAGAAATGCCTGCCGATTCGGGTTACCCCGCTTATCTAGCCGCCCGTTTGGCCTCCTTCTACGAGCGTGCCGGAAAGGTTCGCTGCTTGGGAAGCCCAGAAAGAGAGGGATCCGTCACCATTGTCGGAGCCGTGTCACCTCCTGGTGGAGATTTCTCCGATCCTGTCACGGCCGCCACCCTGGGTATCGTACAGGTGTTTTGGGGTCTCGACAAGAAGCTCGCCCAGCGTAAGCACTTTCCCTCCATCAACTGGCTCATCTCTTACAGTAAGTACATGAGAGCCCTCGAGGACTTCTACGAGAAGAGCTACCCCGAGTTTATCGCCCTCCGTACCAAGTGCAAAGAGATTCTCCAGGAAGAAGAAGATCTTTCCGAAATCGTGCAGCTGGTCGGTAAAGCCTCGCTCGCCGAGTCTGACAAGATCACGCTTGAGGTGGCCAAGATCATCAAGGACGACTTTTTGCAGCAGAACGGATACACGCCTTATGATCGTTTCTGCCCATTCTACAAGACCGTCGGCATGCTCAAGAACATGATTGCTTTCTATGATTTGGCCCGCCATTCGGTCGAATCGACCGCCCAATCAGAGAACAAGGTCACATGGGCTATCATCAGGGATCACATGGGAGACCTCATCTACCAACTATCTGCCATGAAATTCAAGGACCCACAAAAGGACGGTGAGGAGAAGATCAAGAAGGATTACGACGACTTGCTGGAGGCTATGCAGAACGCCTTCAGGAACCTCGAAGACTAA

>ubiquitin C variant (UbiQ)

TGGAAAAACCATCACTCTGGAGGTTGAGCCTTCGGACACAATTGAAAATGTTAAAGCCAAGATCCAGGACAAGGAGGGCATTCCCCCAGATCAGCAGAGGTTGATCTTTGCTGGCAAACAATTGGAAGATGGACGCACCCTCTCTGACTACAATATTCAAAAGGAATCCACCCTACACTTGGTTTTGAGGCTAAGAGGAGGAATGCAAATTTTCGTCAAAACTCTAACTGGAAAAACCATCACTCTGGAGGTTGAGCCTTCGGACACAATTGAAAATGTTAAAGCCAAGATCCAGGACAAGGACGGCATTCCCCCAGACCAGCAGAGGTTGATTTTCGCCGGCAAACAATTGGAAGACGGCCGCACTTTGTCAGATTACAACATTCAAAAGGAATCCACTCTTCACTTGGTGCTAAGATTGAGAGGAGGCATTCAGATTTTTGTTAAGACTTTGACTTGCAAGACCATCACTCTGGAAGTTGAACCCTCCGACACTATTGA

>glyceraldehyde-3-phosphate dehydrogenase (GAPDH)

TGGACAAGTACAACCCAGCTGATAAGGTCATCACCAATGCTTCCTGCACAACCAACTGCTTGGCACCTTTGGCCAAGGTCATACACGACAACTTCGAGATCATCGAAGGTTTGATGACCACCGTGCACGCCACCACTGCCACCCAGAAGACTGTTGACGGTCCATCTGGCAAATTCTGGAGAGATGGACGTGGAGCCCAACAAAACCTGATCCCCGCCGCCACTGGAGCTGCCAACGCCGTCGGAAAGGTTATTCCTTCCCTGAACGGAAAATTGACCGGAATGGCTTTCAGAGAGCCAGATGCCAACGATTCCGTTGTTGACATGACCGCTCGTCTTTGCAAGGGTGCATCTTACGATGAAATCAAGGCCAAGATCAAGGAAGCCGCAGAAGGACCTTTGAAGGGAATCTTGTGTTACACCGAGGAACAATTCGTCTCCTCTGACTTCATCATCGAAACTCACAGCTCGGTGTTCGACGCTGCCGCCGGCATATCCCAAAATAACAAATTTTTGAAACTGAACTCTTGGTACGACAAC

>arginine kinase isoform X1 (ArgK)

ATGGTTGACGCTGCAGTTCTTGAGAAACTTGAAGCCGGCTTCAAGAAACTTGAAGGTTCCGACTCCAAGTCCCTCCTGAAGAAATACCTGACCAGGGATGTTTTCGACAAGCTGAAGACCAAGAAGACTTCTTTCGGCTCCACCCTTTTGGATGTCATCCAATCAGGTGTTGAGAATCCTGACTCTGGAATTGGTATCTACGCTCCTGATGCTGAGGCCTACTCCGTCTTCTCTGACTTGTTCGATCCCATCATCGAAGACTACCATAAAGGATTCGGAAGGAATGACAAGCACCCCCCTAAGAACTGGGGTGATGTCAGCGTTTTCGGCAACTTGGACCCTGCTAGTGAATACGTTGTGTCCACCCGTGTCAGGTGCGGTAGATCATTGGAAGGCTACCCATTCAACCCTTGCTTAACCGAAGAGCAGTACAAAGAAATGGAACAGAAAGTTTCCAGCACTTTGTCCGGATTGGAGGGTGAATTGAAGGGTACCTTCTACCCCTTGACTGGAATGAGCAAAGAAGTTCAGCAAAAATTGATTGATGATCACTTCCTCTTCAAGGAAGGTGACAGATTCTTGCAAGCTGCCAACGCTTGCCGTTTCTGGCCCACTGGACGTGGTATCTTCCACAATGAGAACAAAACCTTCTTGGTCTGGTGCAATGAAGAAGATCATCTTCGTCTCATTTCCATGCAGATGGGAGGTGACTTGGGAGAGGTCTACCGTCGTCTTGTAACTGCTGTCAATGACATCGAAAAGCGTTTGCCTTTCTCCCACCACGACAGGCTTGGTTTCCTTACTTTCTGCCCTACCAACTTGGGTACTACTGTCCGTGCTTCTGTGCACATTAAGGTACCAAAACTCGCTGCCAACAAGGCTAAACTAGAGGAAGTTGCTGCCAAATACAACTTGCAAGTACGTGGAACCCGTGGTGAGCACACCGAAGCTGAAGGTGGCATCTACGATATTTCCAACAAGAGGCGTATGGGTCTCACTGAGTTCGAAGCTGTCAAGGAAATGTACGATGGTATTGCCGAACTGATCAAAATTGAGAGGGAATTGTAA

>ribosomal protein S3 (RPS3)

ATGGCTACCACAATTTCAAAGAAGCGAAAATTTGTTGGAGACGGTGTCTTCAAAGCTGAATTAAATGAATTCTTGACCAGAGAACTATCAGAAGATGGGTATTCTGGTGTTGAAGTACGTGTTACCCCAACACGTACTGAAATTATCATTATGGCCACCCGAACGGACCGTGTTCTTGGTGAAAAGAACAGAAGAATCAGGGAATTGACATCTGTAGTTCAAAAACGGTTCAACTTTCCCGAAAACTCCGTTGTTTTGTATGGAGAGAAGGTAGTCAACAGAGGTCTGTGTGCCATCGCTCAAGCTGAATCTTTAAGATTCAAATTGATCGGAGGCCTGGCAGTTAGGAGGGCTTGCTATGGTGTACTTCGTTACATTATGGAATGTGGAGCTAAGGGTTGTGAGGTGGTAGTGTCAGGAAAACTCAGAGGACAGAGAGCAAAGTCAATGAAGTTTGTGGATGGACTTATGATTCACTCTGGTGATCCTTGCAATGAATATGTGGATACTGCCACAAGGCATGTGCTGCTAAGACAAGGAGTTTTGGGAATCAATGTTAAAATTATGTTGCCTTATGATTCTACTGGTAAAATGGGTCCTAAGAGGCCATTGCCAGACAATGTCTCGGTGGTGGAGCCCAAGGAGGAAGTTATTCCGACTCTTCCGAGTTCAGAGATAAAAGCTGATGTTCTGGCACCCATTTAA

>heat shock protein 83 (HSP83)

TTAATCTACTTCTTCCATTCGGGAAGCGTCTTCGGCGTCTCCTCCTTCGGCGGGTGCCTCAGCAGCAGGAGCGTCATCAGTCAACATAACTTCCTCCTCGTCGATGCCCAAACCAAGTTTGATCATTCGGTAGATTCGAGAGGCGTGAACTTGAGGTTCTTCGAGAGTAAAGCCGGAACTGAGGAGGGCAGTCTCGAACAACAAGATGACCAAGTCTTTAACAGCTTTGTCGTTCTTGTCAGCCTCCGCTTTCTGGCGTAGGTTTTCAATGATTGGATGATCGGGATTGATTTCCAAGTGCTTTTTGGCGGACATGTAACCCATTGTGGATGTGTCACGCAGAGCTTGAGCTTTCATGATACGTTCCATATTGGCAGTCCATCCGTACTGCGAGGTGACAATACAGCACGGTGACTCTACGAGCCTGTTGGACACCACCACCTTCTCGACTTTGTTGTCCAGAATGCTCTTCATTACTTTGCACAAGCCTTCGAATTTGGTCTTGTCCTCTTCGCGTTTTTTCTTCTCTTCTTCAGTTTCTGGCAGTTCCAAGCCTTCTTTGGTAACTGAGACAACTTGTTTGCCATCATATTCTTTGAGGTGTTGGACAACATACTCATCAATGGGTTCAGTCATGTAGACAACTTCAAATCCACGCTTCTTGACTCGTTCAACAAAAGCAGAGTTGGCTACTTGTTCTTTGCTTTCACCCGTAATGTAGTAGATGGATTTCTGGTTCTCCTTCATGCGGCTGACGTATTCCTTAAGTGAGCAGGCTTCATCTCCGCTGGCTGAAGTGTGGTAACGAAGCAAGTCAGCAAGTTTGGCTCGGTTTTGAGTGTCTTCATGGATGCCCAACTTCAAGTTCTTGGAGAATTGTTCGTAGAACTTCTTGTAACCGTCCTTGTCTTCAGACAGTTCTTCAAACAGTTCCATGCATTTCTTCACCAAATTCTTGCGGATCACCTTGAGGATCTTATTCTGTTGCAACATTTCACGAGAAATGTTCAAAGGCAAATCTTCAGAGTCGACCACTCCCTTGATGAAGTTCAAGTATTCAGGGATGAGTTCCTCACAGTTGTCCATGATGAAGACACGTCTGACGTAGAGTTTGATGTTGTTCTTGCGTTTCTTGCTTTCGAAGAGGTCGAATGGCACACGACGAGGCACGAAAAGAAGAGCTCTGAATTCCAACTGGCCTTCAACACTGAAGTGTTTTACGGCTAAGTGATCTTCCCAGTCGTTGGTTAGGGATTTGTAGAATTCACCATATTCTTCCTGGCTGATATCATCAGGGTTTCTGGTCCAGATTGGTTTAGTTTTGTTCAGTTCTTCATCTTCAGTGTATTTCTCTTTGATGGTCTTCTTCTTCTTCTTCTCATCCTTGTCCTTCTTGTCTTCATCCTCATCCTCTCCAATGTCTTCAACTTTTGGTTTGTCGGTGTTTTCATCTTCGGTTTCTTTCTTCTCTTCCTCAGCCTCGTCGTCGCTCAGTTCCTTTTCACGTTCTTTCTCCACAACCAGTTTAATGGGATAACCAATGAACTGGGAGTGTTTCTTGACGATTTCCTTCACTTTGCTCTCCTCCAGGAATTCAGTTTGGTCTTCCTTGATGTGCAAAATGATTTTGGTACCGCGTCCTAAAGGTTCACCCTGGTCTGCACGAATGGTGAAGCTACCACCAGCAGAGGATTCCCAAATGTACTGCTCATCGTCGTTGTGTTTGGAGATGACGGTCACCTTGTCGGCGACAAGATAAGCGGAGTAGAAACCCACACCAAACTGACCGATCATGCTAATGTCAGCGCCGGCCTGCAGGGCTTCCATGAAGGCCTTGGTTCCGGATTTGGCGATGGTACCCAAATTGTTCACCAGGTCGGCTTTGGTCATACCAATACCGGTGTCGATGAGGGTTAAGGTGCCCTCGCTCTTGTTGGGGATGATTTTGATGTAGAGTTCCTTGCCCGAGTCCAGTTTGGAGGGGTTGGTGAGAGATTCATAACGGATTTTGTCCAAAGCGTCGGAGGAGTTGGAGATCAATTCTCGGAGGAAAATTTCTTTGTTGGAGTAGAAGGTGTTGATGATCAAGCTCATCAACTGGGCGATTTCAGCCTGGAAGGCGAAGGTTTCAACTTCTTCTGGCAT

> NADH dehydrogenase subunit 1(NADH)

GTGTCCAATAGCGTTCGCTGTATGTGTGTGTGAGTGCAGACCAACTTTGCATTTCAAATGGACGTGATTTCGCAACGCGACTTCGTGCTAGATCGAACGAAGATATTTAGCATAAAGATATGTATTATCCATGTCTGATTTCCTACGAACCTACACGTTTCTTCGGCTCTCGTTCTAAATGAAGATCTATTGAATTCGAAGTATTTTTGTTCGGCGGAAGATTCCTTCAGATCTGATTTACTTTCTTGCTAGCATCGTAAACCTATTATTTATCGAAGAAGCTGAACTTGTACAAAAGAATTATCAGTATTTTAAAATTACCGTCATGAGAAGAAATCATCGATGTGAAAAAAACAAACATAACTATCGCAAGTTTTTCTATTGTTGTGGCAAAAAACTAATAAGCATTACTTGAATCCTTTGAATTATGTAGGGGGTTATTAAAAAGTTTTTTGTGATGGAGGAGGTTCATTGTCAATTTTACACATGTTAATGGCATAATAATATTTCCCAAAAATGAAAATATTACAAGTTTTGAAATTTTGTTATATTGGCAGAGTTAGTGCTATAAATTTAGAATTTATGAACATGAGTTTTTACTTGTATATAATACTTGATTTTAAAAGGTGCTATTTTATTAATTATAGTAATACTAGTTTAATACTAGTTTAATTCAAATTGTATGTGTTTTAGTAGGGGTAGTTTTTTAACTTTATTGGAACGTAAGGTTTTAGGATACATTCATATTCGAAAGGGGCCTAATAAATTAGGTTTTGTAGGGTTACTTCAACCTTTTAGAGATGCTATTAAATTATTTAGAAAAGAACAAACGTTTCCTATTTTTTCTAATTTAGTTATTTATTATCTATCTCCTGTTATAAATTTATTTTTTTCTTTATTATTATGGATAAGTTTTCCTTTTTTTTCAGTTAATTTTATATTTTCTTTTTCTCTTTTATTTGTTTTAAGAATTAGAAGTTTAGGAGTTTATACGGTAATATTAGCT

>60S ribosomal protein L17 (RPL17)

ACTCAGCATTAAAATATCTTTTTTAATGATACGGCTACCTCAGATATATACACGACCAGA

TACACTCTTTCCCTACACGACGCTCTTCCGATCTCGAAGACGTGCACTTCACACTTCACT

TTTCCGTCATCCAAAGCCAAACAACATCTGTCAAAGGTCTTTTCTGTTGAAGCGTTTTGC

TCTTTAGGTGAGAGGTGAGGATCAAAGATGGGTCGGTACGCTAGAGAACCCGAAAATGCCGCCAAGTCCTGCAAAGCTCGCGGCTCCAATTTGAGAGTTCATTTCAAAAACACATGTGAAACTGCAAATGCAATTCGCAAAATGCCACTGAAAAGGGCTCAGGCATACCTCAAGAATGTTGTCGCGATGAAGGAATGTATTCCATTCAGACGTTTTAATGGTGGAGTTGGTAGATGTGCTCAAGCTAAACAGTTTGGAACCACCCAAGGACGATGGCCCAAAAAATCAGCTGAGTTTTTACTCCAACTTTTAAGAAATGCTGAAAGTAATGCTGATTACACTGGTCTTGATGTGGACCGCCTGGTTGTAGACCACATTCAAGTTAACAGAGCAGCATGTTTGAGGCGGCGTACATACAGGGCTCATGGACGGATTAATCCTTACATGTCTTCACCTTGTCATATTGAATTGTGGCTCACTGAAGGTGA
