# Supplementary material for: Reference Gene Selection for Normalizing Gene Expression in Ips Sexdentatus (Coleoptera: Curculionidae: Scolytinae) Under Different Experimental Conditions
Source: Front Physiol. 2021 Oct 27;12:752768. doi: 10.3389/fphys.2021.752768 (PMC8580292; doi:10.3389/fphys.2021.752768)
Supplement: Supplementary file 3 [file Table_3.DOCX]

**Table S3.** Ranking of the candidate reference genes based on stability values performed by Delta Ct, BestKeeper, RefFinder, NormFinder, and geNorm in all tissues stages (Head, Gut, Fat body and WB) of male and female insects.

| **Genes** | **ΔCt Method** | | **Best keeper** | | **RefFinder** | | **NormFinder** | | **geNorm** | | **Recommended Genes** |
| --- | --- | --- | --- | --- | --- | --- | --- | --- | --- | --- | --- |
|  | **Stability** | **Rank** | **Stability** | **Rank** | **Stability** | **Rank** | **Stability** | **Rank** | **Stability** | **Rank** |  |
| *eEF2* | 1.36 | 1 | 1.97 | 6 | 1.57 | 1 | 0.223 | 3 | 0.47 | 5 | *β-Tubulin*, |
| *β-Tubulin* | 1.47 | 2 | 1.93 | 5 | 2.11 | 2 | 0.160 | 1 | 0.46 | 4 | *RPS3* |
| *GAPDH* | 1.51 | 3 | 2.46 | 10 | 4.05 | 4 | 0.197 | 2 | 0.4 | 2 | and *eEF2* |
| *RPS3* | 1.55 | 4 | 1.56 | 2 | 3.76 | 3 | 0.243 | 4 | 0.4 | 1 |  |
| *NADH* | 1.58 | 5 | 1.75 | 4 | 4.23 | 5 | 0.333 | 12 | 0.67 | 10 |  |
| *HSP83* | 1.72 | 6 | 2.09 | 9 | 6.64 | 7 | 0.279 | 10 | 0.71 | 11 |  |
| *UbiQ* | 1.8 | 7 | 1.21 | 1 | 4.3 | 6 | 0.267 | 8 | 0.55 | 7 |  |
| *V-ATPase-A* | 1.87 | 8 | 2.07 | 8 | 8.24 | 9 | 0.258 | 5 | 0.45 | 3 |  |
| *RPL17* | 1.88 | 9 | 1.73 | 3 | 6.82 | 8 | 0.312 | 11 | 0.74 | 12 |  |
| *ArgK* | 1.9 | 10 | 1.99 | 7 | 8.91 | 10 | 0.260 | 7 | 0.51 | 6 |  |
| *Myosin-L* | 2.06 | 11 | 3.15 | 12 | 11.24 | 11 | 0.259 | 6 | 0.63 | 9 |  |
| *Actin* | 2.17 | 12 | 2.84 | 11 | 11.74 | 12 | 0.275 | 9 | 0.6 | 8 |  |
