# Supplementary figures and images for: Reference Gene Selection for Normalizing Gene Expression in Ips Sexdentatus (Coleoptera: Curculionidae: Scolytinae) Under Different Experimental Conditions
Source: Front Physiol. 2021 Oct 27;12:752768. doi: 10.3389/fphys.2021.752768 (PMC8580292; doi:10.3389/fphys.2021.752768)

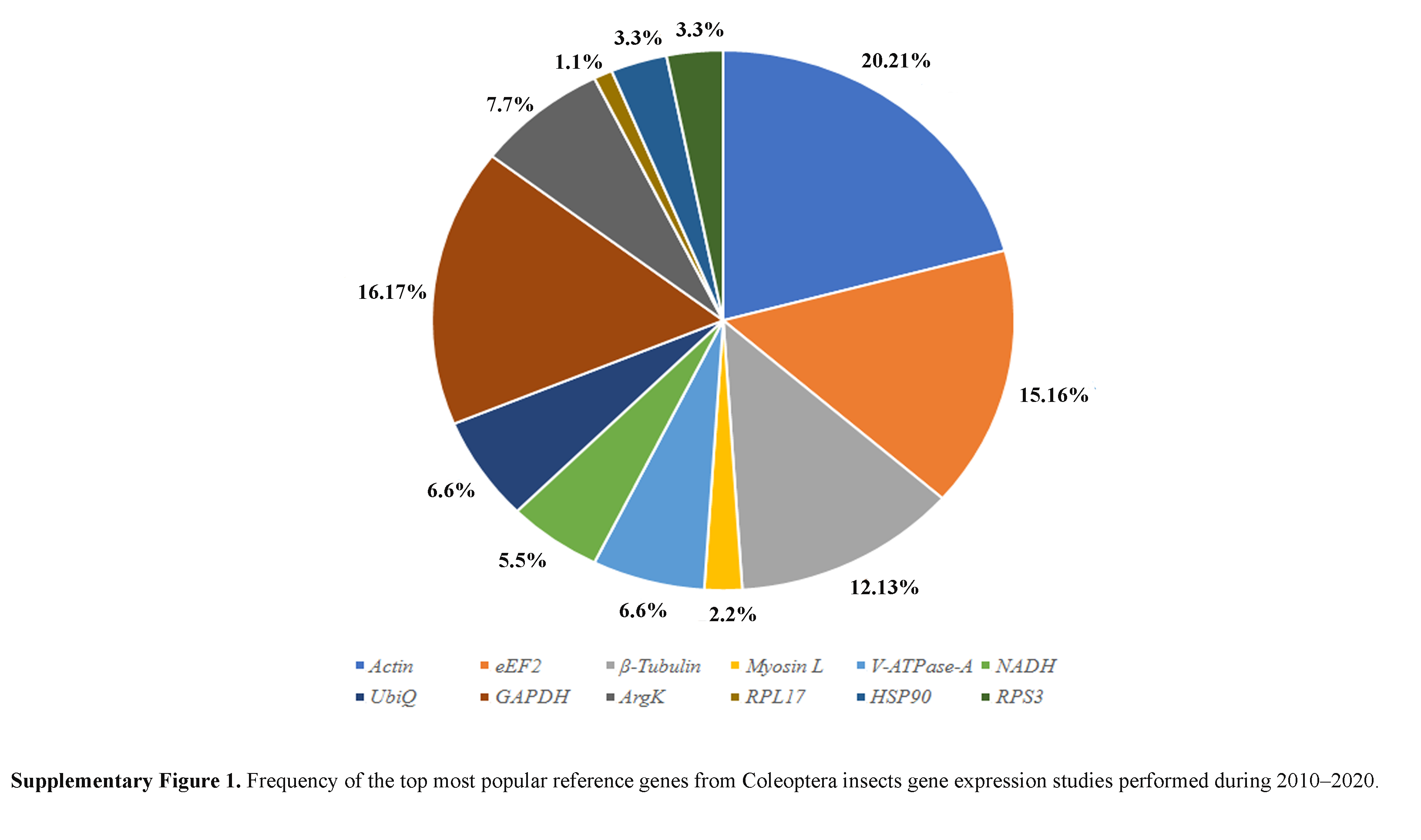

Supplement: Supplementary file 4 [file Image_1.TIF]

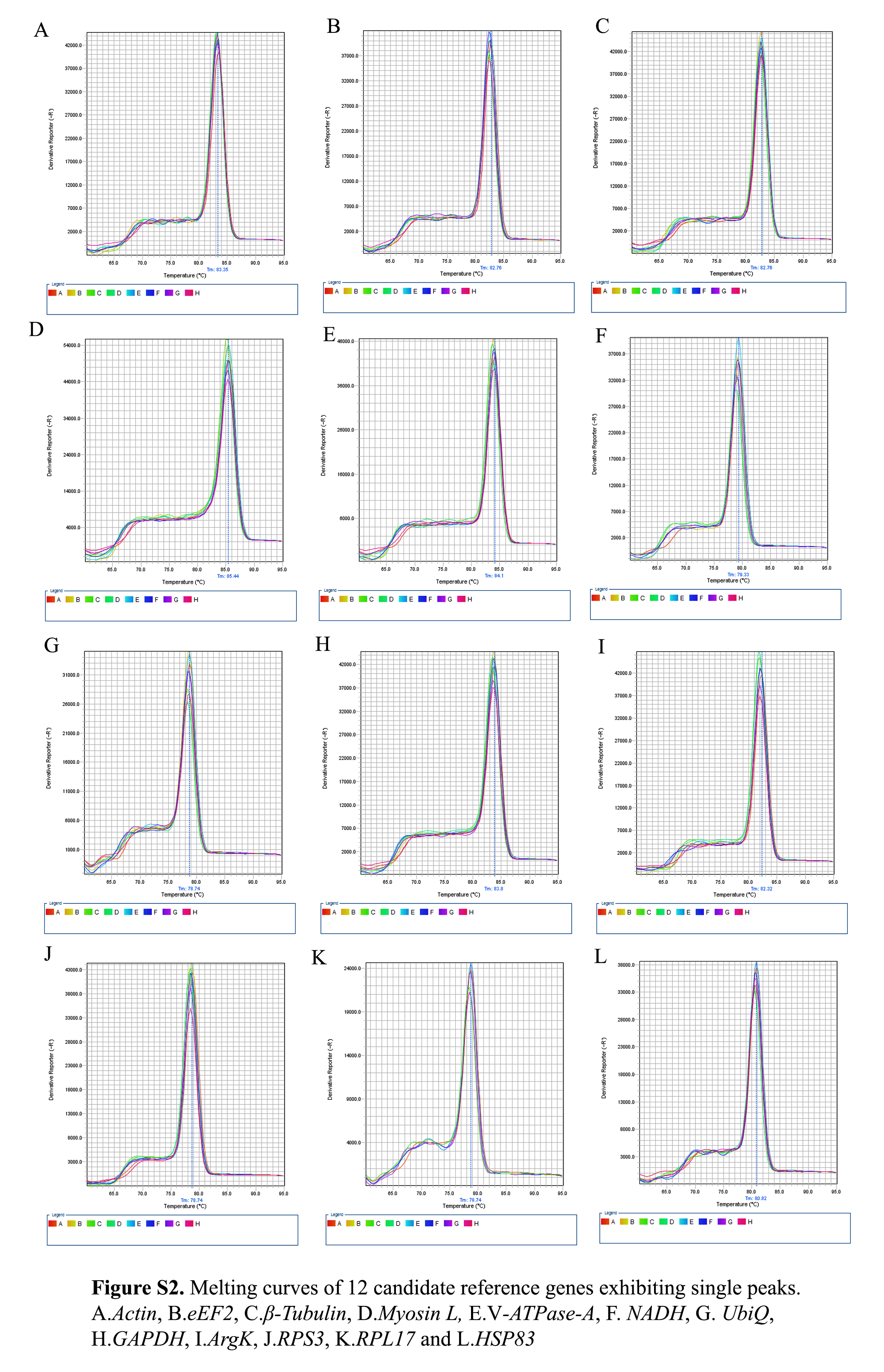

Supplement: Supplementary file 5 [file Image_2.TIF]

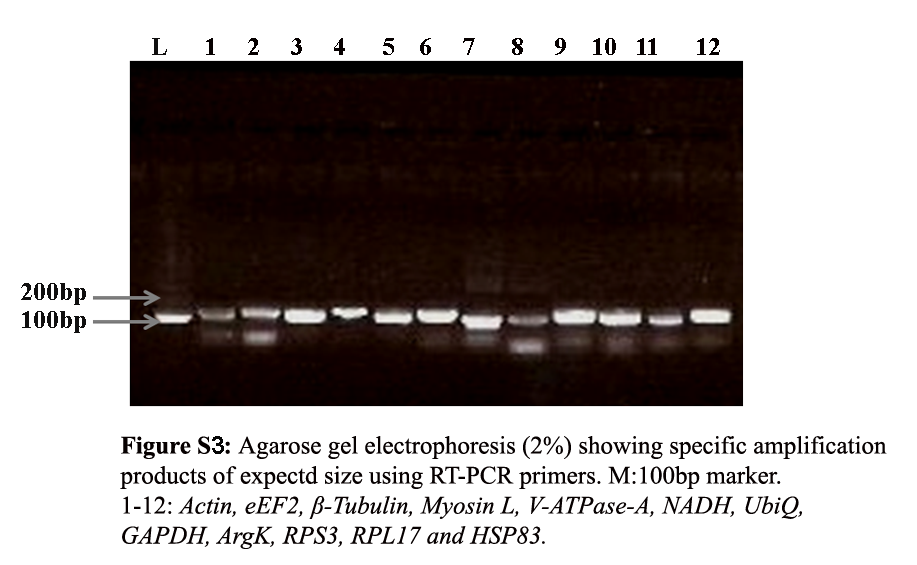

Supplement: Supplementary file 6 [file Image_3.TIF]
